# Nodal signaling establishes a competency window for stochastic cell fate switching

**DOI:** 10.1016/j.devcel.2022.11.008

**Published:** 2022-12-05

**Authors:** Andrew D. Economou, Luca Guglielmi, Philip East, Caroline S. Hill

**Affiliations:** 1 Developmental Signalling Laboratory, The Francis Crick Institute, London, UK; 2 Bioinformatics and Biostatistics Facility, The Francis Crick Institute, London, UK

## Abstract

Specification of the germ layers by Nodal signaling has long been regarded as an archetype of how graded morphogens induce different cell fates. However, this deterministic model cannot explain why only a subset of cells at the early zebrafish embryo margin adopt the endodermal fate, whereas their immediate neighbours, experiencing a similar signaling environment, become mesoderm. Combining pharmacology, quantitative imaging and single cell transcriptomics, we demonstrate that sustained Nodal signaling establishes a bipotential progenitor state from which cells can switch to an endodermal fate or differentiate into mesoderm. Switching is a random event, the likelihood of which is modulated by Fgf signaling. This inherently imprecise mechanism nevertheless leads to robust endoderm formation because of buffering at later stages. Thus, in contrast to previous deterministic models of morphogen action, Nodal signaling establishes a temporal window when cells are competent to undergo a stochastic cell fate switch, rather than determining fate itself.

## Graphical abstract

**Graphic abstract:**
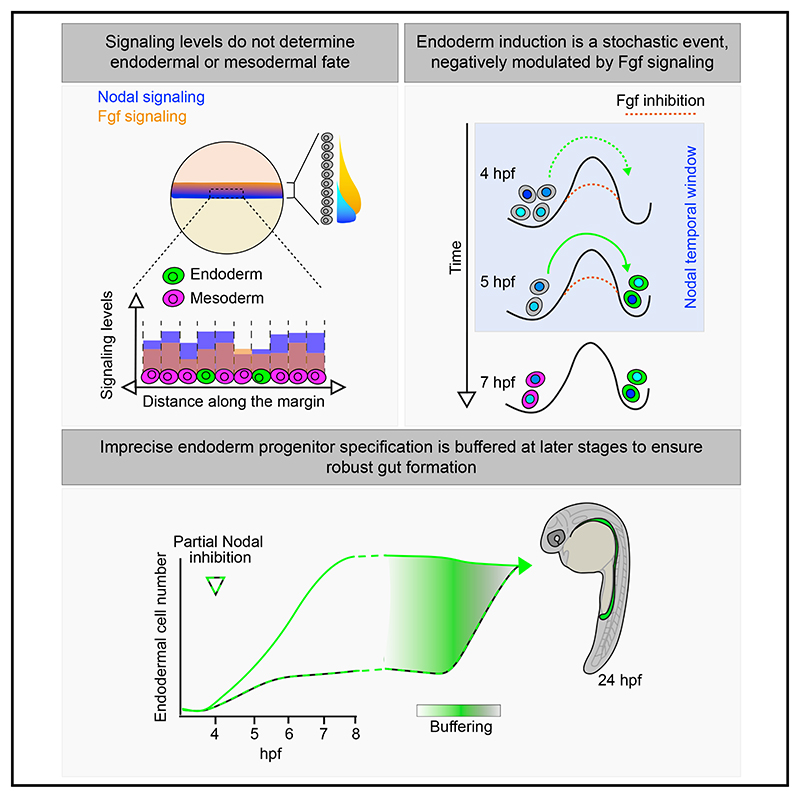

## Introduction

During embryonic development, pluripotent cells are progressively guided to a variety of cell fates by a series of decision-making processes involving chemical and mechanical signals. Crucially, these different cell types must be specified at the correct time and location. One of the most influential principles for how this happens has been the patterning of embryos by morphogen gradients. In its simplest form, a morphogen is produced at a localized source and diffuses across a tissue to form a gradient, with cells in different positions being exposed to different levels of the morphogen and consequently adopting different fates. Since it was originally proposed by Lewis Wolpert^1^, numerous examples of potential morphogens have been identified across a diversity of developmental systems. These studies have resulted in several variants on the mode of morphogen gradient function, such as temporal signal integration, and the importance of downstream transcriptional circuits in morphogen interpretation.^2–5^ However, the importance of the amount of morphogen exposure in ultimately determining cell fate has remained a major theme in developmental biology.

One of the best-known examples of a morphogen in vertebrate development is the role of the transforming growth factor b (TGF-b) family member Nodal in mesoderm and endoderm specification in early zebrafish development.^6–8^ Two Nodal ligands, Nodal-related 1 and 2 (Ndr1/2), are expressed in the early zebrafish embryo, where they signal through a serine/threonine kinase receptor complex comprising two type I receptors, two type II receptors, and the co-receptor Tdgf1 (also called One-eyed pinhead (Oep)).^6^ Upon ligand binding, the activated receptors phosphorylate receptor-regulated Smads (Smad2 in the early zebrafish embryo), allowing them to complex with Smad4 and accumulate in the nucleus. Together with additional transcription factors such as Foxh1 and Mixl1, these Smad complexes bind to enhancers and initiate a new program of gene expression.^6^ Nodal signaling plays a key role in the specification of the germ layers, as mutants affecting the ligands, receptors, or Smad2 show a characteristic phenotype which lacks many mesodermal and all endodermal derivatives. These mutant phenotypes are also replicated with small molecule inhibitors of the type I receptor kinase activity.^9–12^

In the early zebrafish embryo, Nodal ligands have been proposed to act via a morphogen gradient to induce the endodermal and mesodermal cell fates.^13^ Endodermal progenitors are initially marked by the expression of *sox32*, a gene encoding a transcription factor that is essential for induction of the endodermal lineage. Embryos null for *sox32* (the *casanova* mutant) make no endoderm.^14^ The endodermal progenitors are found in the cell tiers closest to the embryonic margin, whereas well-established mesodermal markers such as *tbxta* are expressed up to 10 cell tiers from the embryonic margin.^3,15–17^ As the ligands Ndr1/2 are initially expressed at the margin in an extraem-bryonic tissue called the yolk syncytial layer (YSL) during early epiboly stages, it was proposed that they diffuse to form a gradient towards the animal pole.^15,18,19^ In the cells closest to the source (the YSL) where ligand levels are assumed to be Published by Elsevier Inc. highest, endoderm is induced, with lower signaling levels more distant from the YSL inducing mesoderm.

However, recent work has suggested that Nodal does not function as a classical morphogen. Direct visualization of the range of Nodal activity revealed that it only extends about five cell tiers and therefore is not able to account for the expression of mesodermal target genes up to 10 cell tiers from the YSL.^20^ Indeed, these long-range mesodermal targets are in fact induced by Fgf signaling through the Ras-Raf-Mek1/2-Erk1/2 branch of the pathway downstream of the receptors, with the Fgf ligands themselves targets of Nodal signaling.^20^ Moreover, Fgf signaling through phosphorylated Erk (P-Erk) was also demonstrated to inhibit endoderm induction, as inhibition of Fgf/P-Erk signaling led to increased numbers of endodermal progenitors.^21^ We also showed that the endoderm fate is restricted to the first two cell tiers by the local suppression of Erk signaling through the dual specificity phosphatase Dusp4, which is itself also a direct Nodal target gene.^21^ The Nodal gradient is temporal as well as spatial, with the cells closest to the YSL that show the highest levels of signaling (read out by levels of phosphorylated Smad2 [P-Smad2]) being those induced for the longest duration.^9,21^ Moreover, sustained Nodal signaling is important for endoderm progenitor specification, as Nodal induction of *sox32* requires a cascade of expression of other Nodal-induced transcription factors—specifically, Tbxta, Tbx16, Mixl1, and Gata5.^16,22–24^ Nodal signaling at the margin occurs in a temporal window of about 2 hours from approximately 4 hours post-fertilization (hpf) (sphere stage) to approximately 6 hpf (shield stage). It is shut off by the expression of the Nodal antagonists Lft1/2 at around 50% epiboly (5.3 hpf), whose translation before this time is inhibited by the action of the microRNA *miR430*.^20^ Thus, Nodal signaling provides a temporal window for the specification of endoderm and mesoderm progenitors.

A striking feature of endoderm induction in zebrafish is that whereas the domains of Nodal and Fgf signaling extend all around the embryonic margin, only a subset of the marginal cells express *sox32* and become endoderm. Lineage tracing shows that the remainder become mesodermal progenitors.^14,17^ Cell tracking has shown that the position of the cells relative to the margin is relatively constant at these early stages^9^ and thus these most marginal cells are presumably all exposed to relatively high levels of Nodal signaling and suppressed Fgf signaling. Why only a subset of the cells exposed to the same inductive signals are induced to the endodermal lineage remains a conundrum. There is no evidence for a pre-pattern within the first two cell tiers, as a similar “salt and pepper” pattern of *sox32* expression was observed in the animal pole when a Nodal-expressing clone was introduced at the 128-cell stage.^21^ Furthermore, the spacing of these *sox32*-positive endoderm progenitors is not the result of other signaling pathways—for example, lateral inhibition through Notch signaling.^16,25^

Here, we investigate how neighbouring cells located in the same region of the embryo and exposed to the same signaling environment are induced to different lineages. Our data do not support the consensus view of morphogen gradients that cell fates are specified by cells reading out different signaling levels. Instead, we demonstrate that the distribution of endodermal progenitors is the result of a stochastic process. We show that Nodal signaling provides a competency window for the stochastic switching of bipotential progenitors to an endodermal fate, with lower levels of Erk signaling favoring the switching process. Cells that do not switch to the endodermal fate differentiate to mesoderm. This raises the question of how such probabilistic switching can accurately regulate the number of progenitors induced. We demonstrate that zebrafish embryos are robust to significant variation in the numbers of early endodermal progenitors, as their numbers are corrected during and after gastrulation to produce viable embryos with the appropriate amount of endoderm.

## Results

### Dynamics of mesoderm and endoderm induction and their separation

In many species, a common progenitor state of mesendoderm has been suggested to give rise to both mesoderm and endoderm.^26^ In morphogen gradient models, high Nodal signaling is thought to induce endoderm, with lower levels of Nodal signaling leading to mesoderm.^27,28^ However, in the zebrafish embryo, mesoderm and endoderm progenitors arise in a “salt and pepper” pattern in the first two cell tiers from the embryonic margin in cells that are apparently exposed to similar levels of both Nodal and Fgf signaling. To investigate the underlying mechanism, we first set out to establish the dynamics of mesoderm and endoderm induction.

**Figure 1:**
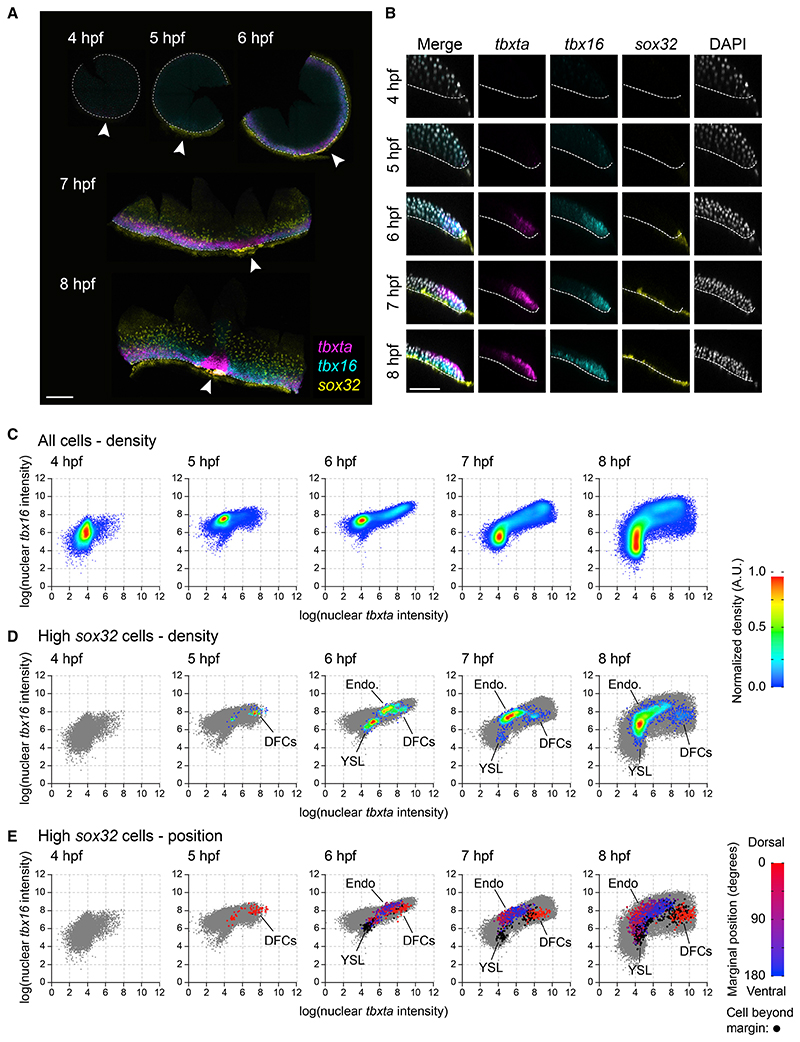
*sox32*-expressing endoderm progenitors emerge from a population of *tbxta/tbx16*-positive progenitors (A) Time series showing maximum intensity projections (MIP) of RNAscope for *tbxta*, *tbx16* and *sox32*. Dashed white line, embryo margin; arrowhead, dorsal. (B) Z-reconstruction through the lateral regions of the embryos in (A). Dashed line marks boundary between embryo and YSL. Colors as in (A); nuclei marked with DAPI (gray). (C) Density scatter plots showing nuclear intensity staining for *tbxta* and *tbx16* for all cells pooled from four embryos for each time point. (D) As in (C), but showing cells with elevated *sox32* expression (defined as log[nuclear sox32 intensity] > 8.5) in the context of all cells (gray). The density scale in (C) and (D) is normalized to a maximum density of 1.0 for each plot. (E) Scatter plots from (D) colored to show the position of the closest point at the margin of the embryo from dorsal (0 degrees: red) to ventral (180 degrees: blue). Lateral regions on both sides of each embryo are given the same positional value. Nuclei beyond the margin are marked in black. Populations of cells in (D) and (E) composed predominantly of black cells or red cells are YSL and dorsal forerunner (DFCs), respectively. The remaining cells are endodermal progenitors (Endo). See also Figure S1. Scale bars: 250 μM (A), 100 μM (B).

We collected embryos throughout early development (at hourly intervals from 4-8 hpf) and performed RNAscope *in situ* hybridization for *sox32* multiplexed with two well-established mesodermal markers: *tbxta* and *tbx16*^29,30^(Figures 1A and 1B). We developed an *in toto* quantitative imaging pipeline whereby we segmented all the nuclei in each embryo and quantified the staining intensity for the three markers (Figures 1C and 1D). Tracking the relative expression of *tbxta* and *tbx16* revealed that, by 6 hpf, both markers are expressed around the margin before separating at gastrulation when *tbxta* is expressed alone in an internalized dorsal domain (the future axial mesoderm), with *tbx16* internalized in ventrolateral cells (part of the future paraxial mesoderm) (Figures 1A–C, S1A, and S1B). A domain of non-internalized cells coexpressing the two mesodermal markers is also maintained in the cells closest to the embryonic margin (Figures 1B and S1A–S1C). These results suggest that the most marginal cells—where endodermal progenitors are known to be induced^14^—may already express mesodermal markers (Figures 1A and 1B).

Plotting the levels of *tbxta* and *tbx16* for only the cells with the highest levels of *sox32* expression confirmed that endodermal progenitors do not begin to appear until after 5 hpf (at this time, the only cells with elevated *sox32* were the YSL and the dorsal forerunner cells [DFCs], which are the precursors of Kupffer’s vesicle^14^) (Figure 1D). Moreover, at 6 hpf, endodermal progenitors are found among the most marginal cells with elevated *tbxta* and *tbx16* (Figures 1D and S1C). These cells then internalize and migrate away from the margin (Figures 1B, S1A, and S1B). Of note, *tbxta* levels decrease in most endodermal progenitors from 7 hpf, when they have begun to ingress (although *tbxta* levels remain high in the DFCs) (Figures 1D and 1E). In contrast, there is a broad range of *tbx16* expression levels among the endodermal progenitors (Figures 1D and 1E). Therefore, endodermal progenitors are induced among the population of marginal cells that express mesodermal markers.

### Endoderm progenitors appear randomly without spatial or temporal bias

**Figure 2:**
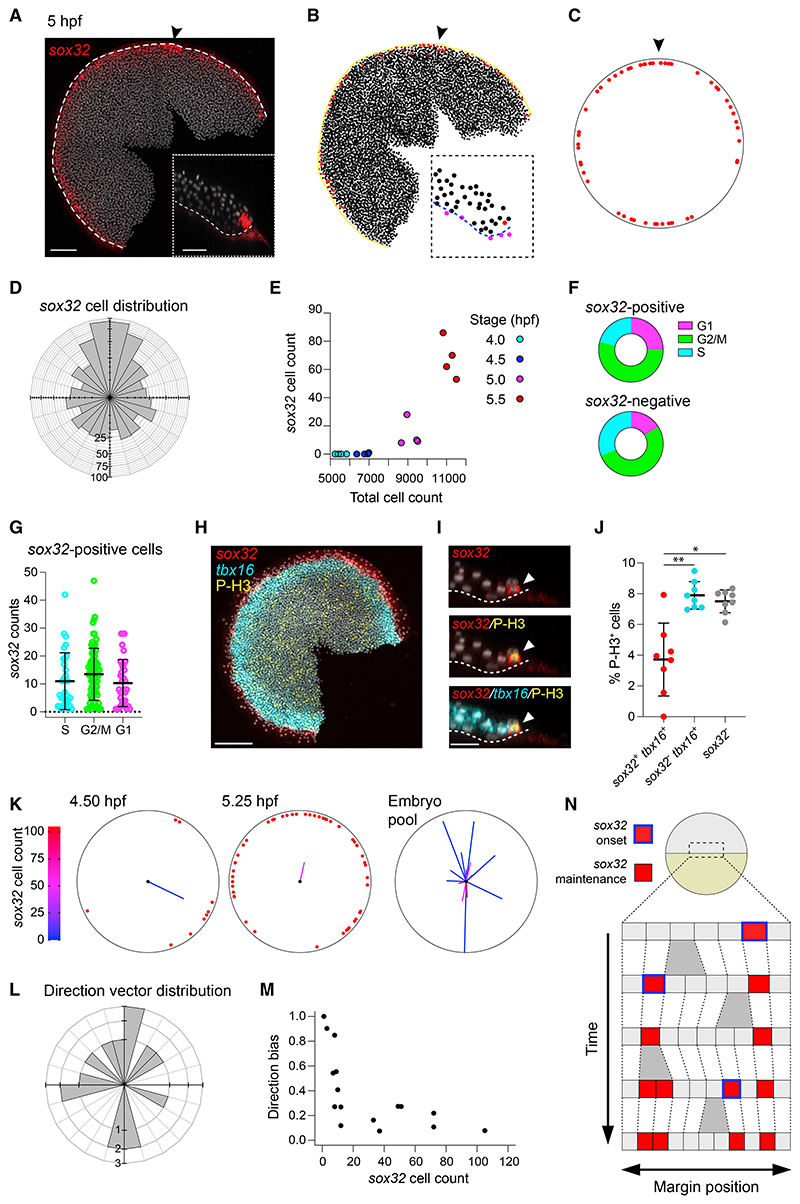
*sox32*-positive cells are induced randomly within the first two cell tiers (A) MIP of RNAscope for *sox32* in a 5 hpf embryo. Nuclei marked with DAPI (gray). Dashed white line, embryo margin; arrowhead, dorsal. Inset: Z-reconstruction through the lateral regions of embryo. (B) Digitization of embryo in (A) showing the centroids from segmentation of all nuclei in the embryo. *sox32*-positive cells are marked in red. Yellow line, embryo margin; arrowhead, dorsal. Inset: Positions of all nuclear centroids from inset in (A). YSL and cells beyond the margin, and thus eliminated from the analysis, are labeled in magenta. Blue dashed line, embryo margin. (C) Reconstruction of embryo in (B) showing the position of *sox32-*positive cells (red). Arrowhead, dorsal. (D) Distribution of 827 *sox32*-positive cells around the margin for 20 embryos collected from 4.25 to 5.25 hpf. Note that dorsal enrichment corresponds to DFCs. Dorsal, up. (E) Relationship between *sox32*-positive cells (YSL and DFCs removed) and total cell number for embryos collected at 30 min intervals from a single clutch. Colors indicate embryonic stage. (F) Donut graphs from the scRNA-seq dataset showing the percentage of cells in G1, G2/M or S phase for *sox32*-positive cells (above) and *sox32*-negative cells (below). (G) Scatterplot from the scRNA-seq dataset showing *sox32* expression levels in *sox32*-positive cells falling in the different phases of the cell cycle. Means ± SD are shown. Dotted line shows zero. (H) MIP of RNAscope for *sox32* (red) and *tbx16* (cyan) combined with immunofluorescence (IF) for P-H3 (yellow) in a 5-hpf embryo. Nuclei are marked with DAPI (gray). (I) Z-reconstruction through the lateral regions of the embryo shown in (H). Dashed white line, embryo margin. Arrowhead shows a cell positive for *sox32*, *tbx16*, and P-H3. (J) Scatter dot plot showing percentage of cells staining for P-H3 among cells that are *sox32*^+^ and *tbx16*^+^, *sox32*^-^ and *tbx16*^+^, or *sox32*^-^. Means ± SD are shown. (K) Two representative embryos showing the distribution of *sox32*-positive cells around the margin. Average direction vector shown on each embryo. Length indicates direction bias and color indicates the total number of *sox32*-positive cells. Embryo pool shows vectors from the 20 embryos in (D) (only 16 vectors are shown, as the four youngest embryos have no progenitors). Dorsal, up. (L) Distribution of average direction vectors for the 16 embryos in (K), grouped into 15-degree bins. Dorsal, up. (M) Plot of direction bias against total number of *sox32*-positive endodermal progenitors for the 16 embryos in (K). (N) Proposed model for the appearance of *sox32*-positive endodermal progenitors. A series of cells at the margin of the embryo proliferate through time (gray bifurcations). In a random manner, cells turn on *sox32* (blue outlined cells), and expression is maintained (red cells). See also Figure S2. Scale bars: 125 μM (A), 50 μM (A, inset), 200 μM (H), 28 μM (I). *p < 0.05; **p < 0.01.

We next determined precisely where endodermal progenitors are located—both relative to their position around the margin and to each other—and how this pattern is generated. We densely sampled embryos across early epiboly (fish were spawned continuously for 1 h, then embryos were sampled every 15 min from 4.25–5.25 hpf) and we used our quantitative imaging pipeline to identify endodermal progenitors as cells with the highest levels of nuclear *sox32* staining and mapped their position around the margin of the embryo, excluding nuclei corresponding to the YSL (Figure 2A–2D and S2A). The dorsal enrichment of *sox32*-positive cells corresponds to DFCs, which were also removed from endodermal cell counts.

We then determined when the endoderm progenitors first appear. Collecting embryos at 30-min intervals from a single synchronized clutch from 4–5.5 hpf, we found that rather than all appearing at once, the number of endodermal progenitors increased steadily through time (Figure 2E). We noticed the same overall trend in the number of *sox32*-positive endodermal progenitors in the mass-spawned embryo dataset described above, although in this case the data were noisier, as they came from many different clutches and the staging was less accurate (Figure S2B). At these embryonic stages, the cell cycle duration is 30–45 min,^31^ meaning that most cells only divide once in the 1.5-h window we are studying. We therefore reasoned that the increase in endodermal progenitors over time could either be the result of progenitors being continuously induced throughout early epiboly, or a small number of progenitors induced during a short, early time window before proliferating at an increased rate relative to their neighbors.

To distinguish between these two scenarios, we investigated expression of cell cycle markers at the margin. We analyzed a published scRNA-seq dataset for 50% epiboly zebrafish embryos^32^ and extracted cells positive for *gata5*, which is expressed in the first two cell tiers from the margin (Figures S2C and S2D and Data S1). Notably, cells within this pool were not biased towards a specific phase of the cell cycle (Figure S2E). Confirming this, the proportion of cells expressing G2/M markers in cells negative or positive for *sox32* was also equivalent (Figure 2F). Moreover, compared to classic G2/M markers, expression levels for *sox32* were uniform irrespective of the phase of the cell cycle (Figures 2G and S2F). To further validate these findings, we stained 5-hpf embryos for phosphorylated histone H3 (P-H3), which marks cells undergoing mitosis,^33^ in combination with *sox32* and *tbx16* (Figures 2H and 2I). The proportion of *sox32*-positive cells expressing P-H3 was not higher, but in fact was lower and more variable when compared with the proportion of *tbx16*-positive cells expressing P-H3 or compared with all the other cells in the embryo (Figure 2J). We thus excluded an increase in proliferation of endodermal progenitors as a mechanism to explain the steady increase in progenitor numbers over time. Instead, we conclude that they are continuously induced throughout early epiboly.

We then focused on how the endoderm progenitors are induced spatially. Pooling all endodermal progenitors from our densely sampled mass-spawned dataset described above, we could not distinguish the distribution of progenitors around the margin from a uniform distribution once the DFCs had been excluded (Figures S2G and S2H). Thus, cells were equally likely to be found at any position around the margin, indicating no bias.

To further explore whether there was any regular pattern in how progenitors were positioned relative to each other, we generated an average progenitor direction vector for each embryo by summing the direction vectors for all endoderm progenitors in an embryo (Figure S2I). Again, we found no directional bias for the orientation between embryos (Figures 2K and 2L). For each embryo, the directional bias in the positions of progenitors was reflected by the magnitude of the direction vector (a large value would reflect all cells being localized in a similar region). We noticed that for embryos with very few progenitors, some had large magnitudes and others small; magnitudes decreased as the numbers of progenitors increased (Figure 2M). We found that all of these features could be recapitulated using an unbiased simulation where, during early epiboly, any marginal cell could turn on *sox32* with a very low probability, and once a cell expressed *sox32*, expression is maintained (Figures 2N and S2J–S2P). The concordance between the simulation and our data indicates that there is no spatial or temporal bias in the induction of endodermal progenitors. Rather, marginal cells turn on and maintain *sox32* expression in a random manner.

### Nodal and Fgf signaling levels affect the likelihood of endoderm induction rather than determining cell fate itself

We next determined what role signaling plays in determining this spatially random pattern of *sox32* expression. To date, differences in morphogen levels have generally been considered at the level of broad graded profiles across fields of cells.^21,34,35^ We considered, however, that the heterogeneity seen in the appearance of endodermal progenitor cells could be the result of cell-to-cell differences in the levels of Nodal or Fgf signaling.

We therefore asked whether the unpredictable nature of *sox32* expression could be explained by noise in cell signaling.

**Figure 3:**
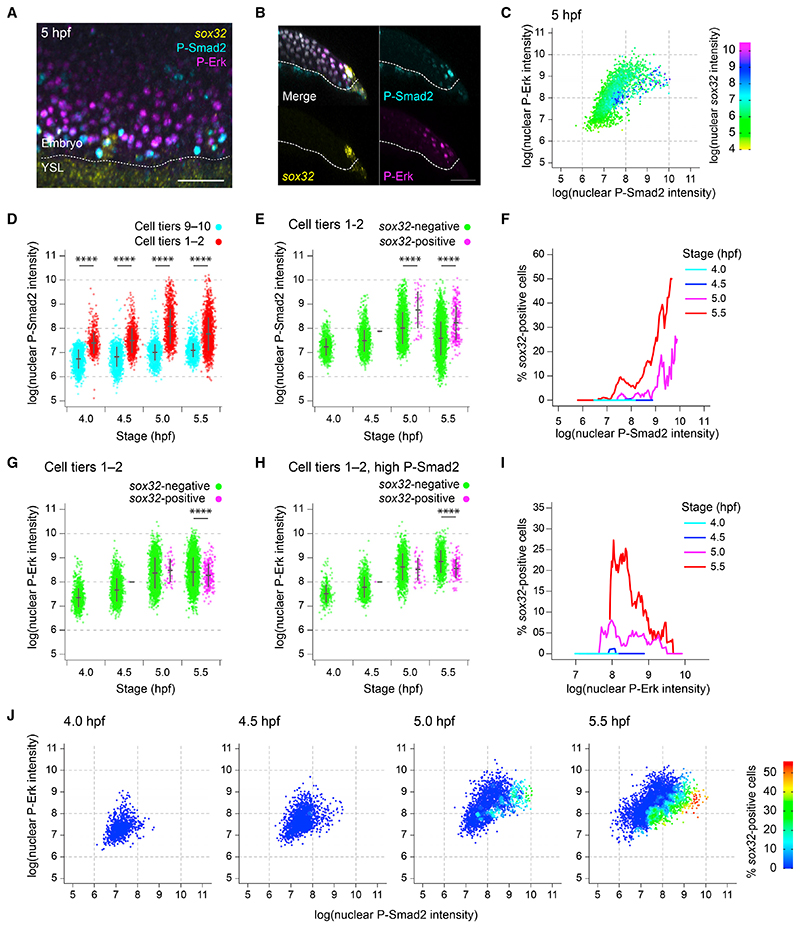
Levels of Nodal and Fgf signaling are not deterministic for endoderm progenitor induction (A) MIP through the margin of a 5-hpf embryo showing IF for P-Smad2 and P-Erk, with RNAscope for *sox32*. Dashed white line, embryo margin. (B) Z-reconstruction through the lateral regions of the embryo in (A) showing *sox32* expression in endodermal progenitors relative to P-Smad2 and P-Erk staining. Dashed white line, embryo margin. (C) Scatter plot showing nuclear staining intensity of P-Smad2 and P-Erk for a single 5-hpf embryo. Cells colored by nuclear staining intensity for *sox32*. (D) Plot showing P-Smad2 staining intensity for all cells in cell tiers 1–2 compared to background (cell tiers 9–10). Means ± SD are shown. (E) Plot showing P-Smad2 staining intensity for all cells in cell tiers 1–2, sorted into *sox32*-positive and -negative cells. Means ± SD are shown. (F) Traces showing the proportion of cells that are *sox32*-positive for a given P-Erk staining intensity for cells in cell tiers 1–2 at different stages. The proportion of *sox32*-positive cells for a given level of P-Smad2 staining is based on all cells within a window of ± 5% of the total range of P-Smad2 staining intensities for that stage. (G) As in (E), but showing P-Erk staining intensity. Means ± SD are shown. (H) As in (G), but showing P-Erk staining intensity for cells with P-Smad2 staining elevated above background levels. Means ± SD are shown. (I) Traces showing the proportion of cells that are *sox32*-positive fora given P-Erk staining intensity for cells in cell tier 1-2 with elevated P-Smad2 staining (defined as in H) at different stages. Traces are as in (F). In (H and I), the 99^th^ percentile of P-Smad2 staining intensity for the cells from the cell tiers 9–10 is used as a threshold to define elevated P-Smad2. (J) Scatter plot showing nuclear staining intensity of P-Smad2 and P-Erk for cells in cell tiers 1-2 through time. Color coding indicates the proportion of cells for the given signaling profile which are *sox32* positive. The data are a re-analysis of the data presented in panels F and I. Analyses in this figure are based on the dataset in Figure 2E. See also Figure S3. Scale bars: 50 μM (A, B). ****p < 0.0001.

We performed a double immunostaining for P-Smad2 and P-Erk (as readouts for Nodal and Fgf signaling through Erk, respectively) simultaneously with an RNAscope *in situ* hybridization for *sox32* (Figures 3A and 3B). By quantifying nuclear levels of *sox32* alongside P-Smad2 and P-Erk for all cells in the embryos, we could profile all *sox32*-positive and *sox32*-negative cells for their Nodal and Fgf signaling states (Figure 3C). We first asked whether the increase in the number of *sox32*-positive cells over time (see Figure 2E) was associated with an overall increase in Nodal signaling levels. Comparing the levels of P-Smad2 across the first two cell tiers (where endodermal progenitors are induced) from 4–5.5 hpf showed that the initial increase in *sox32*-expressing cells at 5 hpf was associated with an increase in mean P-Smad2 levels, but the continued increase in *sox32*-positive cell numbers after this stage (see Figure 2E) occurred without any further increase in overall P-Smad2 levels (Figures 3D and S3A).

Moreover, while levels of P-Smad2 were elevated in the first two cell tiers relative to background, we were struck by how great the range of P-Smad2 levels was among these most marginal cells, with many cells having P-Smad2 levels comparable to background (i.e P-Smad2 levels in cell tiers 9–10^21^). This was not a property of the antibody staining *per se*, as this variation was also seen using subcellular localization of GFP-Smad2 as a Nodal signaling readout,^9^ and the variation was independent of cell depth in the tissue (Figure S3D–S3I). Therefore, it is likely the result of heterogeneity in the responsiveness of cells to Nodal. We tested whether variation in P-Smad2 levels could explain which cells become endodermal progenitors. Comparing P-Smad2 levels between *sox32*-positive and *sox32*-negative cells in the first two cell tiers at 5.0 and 5.5 hpf showed that the mean P-Smad2 levels were indeed significantly higher in *sox32*-positive cells compared to negative cells (Figure 3E; see also Figure S3B). However, there was still considerable overlap in P-Smad2 levels between the two populations (Figures 3E and 3SB).

To better explore the relationship between P-Smad2 and *sox32* expression, we calculated the proportion of cells at different stages that were *sox32*-positive for different levels of P-Smad2. We saw evidence for a temporal effect of P-Smad2, as no *sox32*-positive cells appear before 5 hpf despite moderate levels of P-Smad2 (Figure 3F). Then, at both 5.0 and 5.5 hpf, the proportion of cells that were *sox32* positive increased with the level of nuclear P-Smad2 (Figures 3F and S3C). Strikingly, however, for any given level of nuclear P-Smad2, the number of cells that were *sox32* positive also increased with time. Therefore, while variation in the level of P-Smad2 between cells is predictive of *sox32* expression, it alone is not sufficient to determine whether marginal cells become endodermal progenitors. A given level of P-Smad2 does not determine how many cells will be *sox32* positive as the progenitors accumulate through time; it just makes their appearance more likely.

Given that we and others have previously shown that Fgf signaling inhibits endodermal fate specification,^21,36,37^ we reasoned that cell-to-cell variation in levels of P-Erk within the first two cell tiers might determine whether or not a cell experiencing a given level of nuclear P-Smad2 would express *sox32*. We therefore compared P-Erk levels across cells in the first two cell tiers stratified by *sox32* expression (Figure 3G). At 5.0 and 5.5 hpf, there was substantial overlap in the levels of P-Erk between the two populations, suggesting that variation in Fgf signaling between cells could not explain which cells were induced to become endoderm progenitors. As many of these cells would have low P-Smad2 levels, and are therefore not expected to express *sox32*, we repeated the analysis, restricting it only to cells with elevated P-Smad2. Again, we noted substantial overlap in the levels of P-Erk between *sox32*-positive versus *sox32*-negative cells but found that *sox32*-positive cells at 5.5 hpf had on average lower levels of P-Erk (Figure 3H). We therefore investigated what proportion of the high P-Smad2 cells were *sox32* positive for different levels of P-Erk. Again, at 5.5 hpf we noted that *sox32*-expressing cells were preferentially those with lower levels of P-Erk (Figure 3I). This suggested that, whereas high levels of Nodal and low levels of Fgf signaling did not specifically define the endodermal progenitor population, the signaling levels dictated the likelihood of a cell expressing *sox32*.

To confirm this, we determined the percentage of cells in the first two cell tiers that expressed *sox32* relative to their levels of P-Smad2 and P-Erk. At 5.0 and 5.5 hpf, the percentage of *sox32*-positive cells increased with increasing Nodal and decreasing Fgf signaling, and for a given signaling level, the proportion of *sox32*-positive cells increased with time (Figure 3J).

### sox32 expression, and hence endoderm progenitor induction, is regulated by a bistability

Our data show that *sox32* expression is not a simple readout of heterogeneous signaling inputs. Instead, we hypothesized that *sox32* expression could be bistable, and Nodal signaling may push cells closer to a bifurcation point. Inherent noise in molecular cellular processes would result in some cells by chance crossing this bifurcation point, resulting in the onset of *sox32* expression and irreversibly committing to the endodermal fate. As induction would be a random event in time (and space), *sox32*-positive cells would accumulate through time. In addition, if cells with lower P-Smad2 levels are further from the bifurcation point, fewer endodermal progenitors should accumulate at regions of lower Nodal signaling.

**Figure 4:**
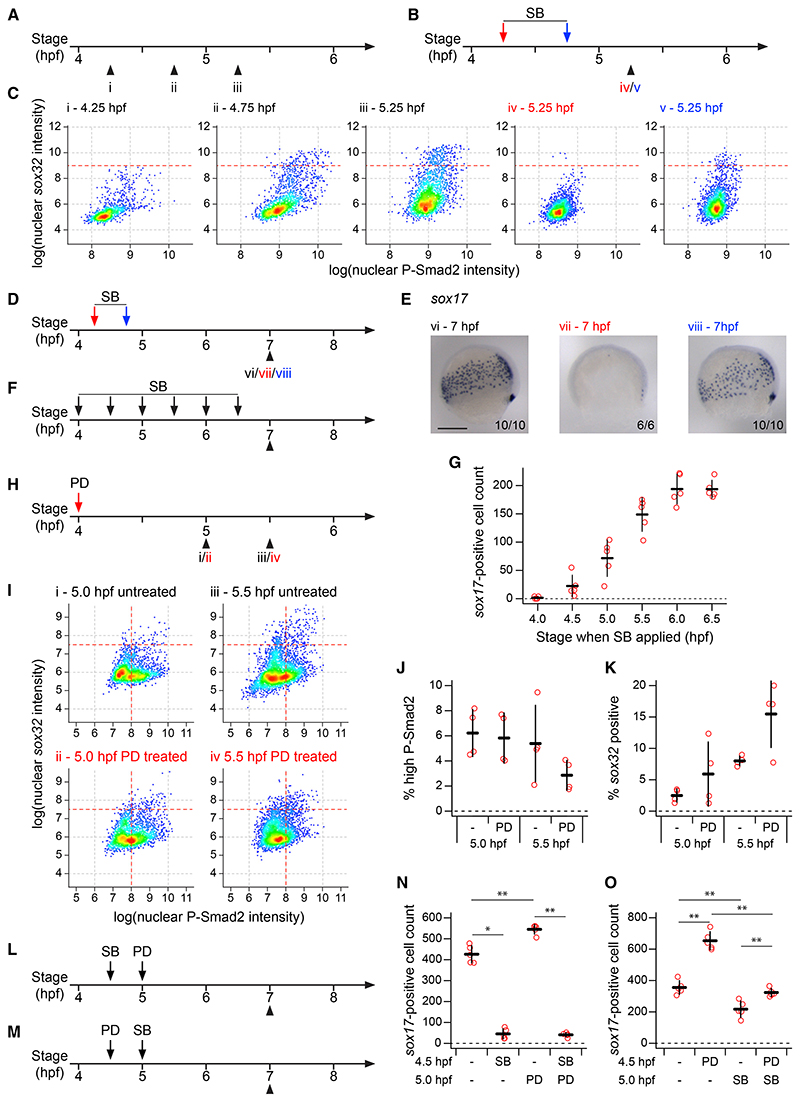
Endoderm progenitors require Nodal signaling for their induction but not maintenance (A–C) Schematics showing collection times of untreated embryos (A) and timings of SB-505124 (SB) application and embryo collection (B) for plots shown in (C). Colors of roman numerals correspond to the treatment (red and blue correspond to arrows in B, black to no SB). Density scatter plots showing nuclear intensity staining for P-Smad2 and *sox32* for all cells in cell tiers 1–2 pooled from four embryos for each condition (C). Dashed red lines indicate threshold defining *sox32*-positive cells. (D and E) As in (B), but showing the timing of SB application and embryo collection for embryos in (E). Colors of roman numerals as in (A) and (B). Representative embryo following WISH for *sox17* (E). Roman numerals refer to the schematics in (D). Dorsal to right; animal pole, top. Numbers in the bottom right refer to the number of embryos showing the phenotype out of the total number studied. (F) As in (B), but showing the timings of SB application and embryo collection for (G). (G) Quantification of *sox17*-positive cell numbers for embryos treated with SB as in (F). Means from five embryos per condition ± SD shown. For comparison, note that for DMSO-treated embryos the mean cell count is 260.8 and the SD, 18.14. (H) As in (B), but showing the timing of PD-0325901 (PD) application and embryo collection for (I). Roman numerals in red indicates PD treatment. (I) As in (C), but Roman numerals correspond to treatments as defined in (H). Horizontal dashed red lines indicate threshold defining *sox32*-positive cells. (J) Plot showing the percentage of cells in (I) with high P-Smad2 (as defined by vertical dashed red lines in (I)) for each treatment condition. Means ± SD are shown. (K) As in (J), but showing the percentage of high P-Smad2 cells in (I) which are *sox32*-positive (as defined by horizontal dashed red lines in I) for each treatment condition. Means ± SD are shown. (L and M) As in (B), but showing the timings of treatment with SB and PD and embryo collection for plots in (N and O). (N and O) Quantification of *sox17*-positive cells number for embryos treated with SB and PD as defined in (L) and (M), respectively. Means ± SD are shown. See also Figure S4. Scale bars: 250 μM (E). *p < 0.05; **p < 0.01

It is well established that Nodal signaling is required for endoderm induction,^14^ and if the system is bistable, endoderm specification should be maintained even if Nodal signaling is withdrawn. We therefore treated embryos with the Nodal signaling inhibitor SB-505124^38^ (see Figure S4A) at 4.75 hpf, when endodermal induction has already begun, collected embryos at 5.25 hpf, and then compared them with DMSO-treated controls. As a control, we also treated embryos at 4.25 hpf, a time before any *sox32*-expressing cells were detectable (Figures 4A–4C). If the Nodal receptor inhibitor was added early, no endodermal progenitors were induced (Figure 4Civ and S4B), but if it was added at 4.75 hpf, then endodermal progenitors were present but in reduced numbers compared with control embryos of the same age treated with DMSO (Figure 4C: compare iii and v; Figure S4B). To fully interpret this result, it was important to demonstrate ongoing transcription in the absence of Nodal signaling. We could still see nuclear *sox32* transcripts in the absence of nuclear P-Smad2 staining (Figures S4A and S4C), suggesting that the maintenance of *sox32* expression in the absence of Nodal signaling is the result of active transcription rather than transcript stability. The instability of the *sox32* transcripts is also supported by the rapid clearance of *sox32* expression after 6 hpf (Figure S4D). Thus, we conclude that Nodal signaling is required for the induction of *sox32*, but not for its maintenance.

At around 5.5 hpf, once embryos have started to gastrulate, endodermal progenitors go on to express *sox17* and then migrate over the yolk towards the dorsal animal region of the embryo.^39^ To confirm that the maintenance of *sox32* expression in the absence of Nodal signaling resulted in cells maintaining their commitment to the endodermal lineage, we repeated the above experiment but fixed embryos at 7 hpf and performed in situ hybridization for *sox17* (Figure 4D). As above, the cells continued to differentiate down the endodermal lineage even in the absence of Nodal signaling (Figures 4D and 4E). This model predicts that, as switching to the endodermal lineage occurs continuously through early epiboly, the later Nodal signaling is inhibited, the more progenitors should accumulate. Repeating the above experiment but applying SB-505124 at successively later timepoints demonstrated that, indeed, the later Nodal signaling was inhibited, the more *sox17*-positive cells were present at 7 hpf (Figures 4F and 4G).

Although Fgf signaling is inhibitory for endoderm induction, it is unclear at what level it inhibits the ability of cells to switch to the endodermal lineage. We reasoned that Fgf signaling could either work downstream of Nodal, by reducing the likelihood of cells switching fate when experiencing a particular level of Nodal signaling, or Fgf could act upstream of Nodal by inhibiting Nodal signaling and thereby indirectly reducing the likelihood of cells acquiring an endodermal fate. To test this, we treated embryos at 4 hpf with the Mek inhibitor PD-0325901^40^ (Figure S4E), collected embryos at 5.0 and 5.5 hpf, and performed immunofluorescence for P-Smad2 with RNAscope for *sox32* (Figures 4H–4K). Upon Mek inhibition, the proportion of cells with elevated P-Smad2 did not increase (Figure 4J), whereas the proportion of these cells which were *sox32* positive did increase (Figure 4K). Therefore, Mek inhibition increases the proportion of cells experiencing a given level of Nodal signaling being induced to endoderm. This indicates that Fgf signaling acts by modulating the likelihood of cells exposed to Nodal signaling acquiring an endodermal fate.

A key prediction of this model is that inhibiting Fgf signaling should only influence endoderm induction if Nodal signaling is active. Indeed, inhibiting Fgf signaling after Nodal signaling had been blocked led to the same number of progenitors as blocking Nodal signaling alone early in development, whilst blocking Fgf alone later in development led to a slight increase in progenitor numbers (Figures 4L and 4N). Conversely, inhibition of Nodal signaling after Fgf signaling inhibition still led to a reduction in numbers of *sox17*-positive cells compared to only blocking Fgf signaling early (Figures 4M and 4O). These observations support the idea that Fgf signaling functions by modulating the effect of Nodal signaling.

**Figure 5:**
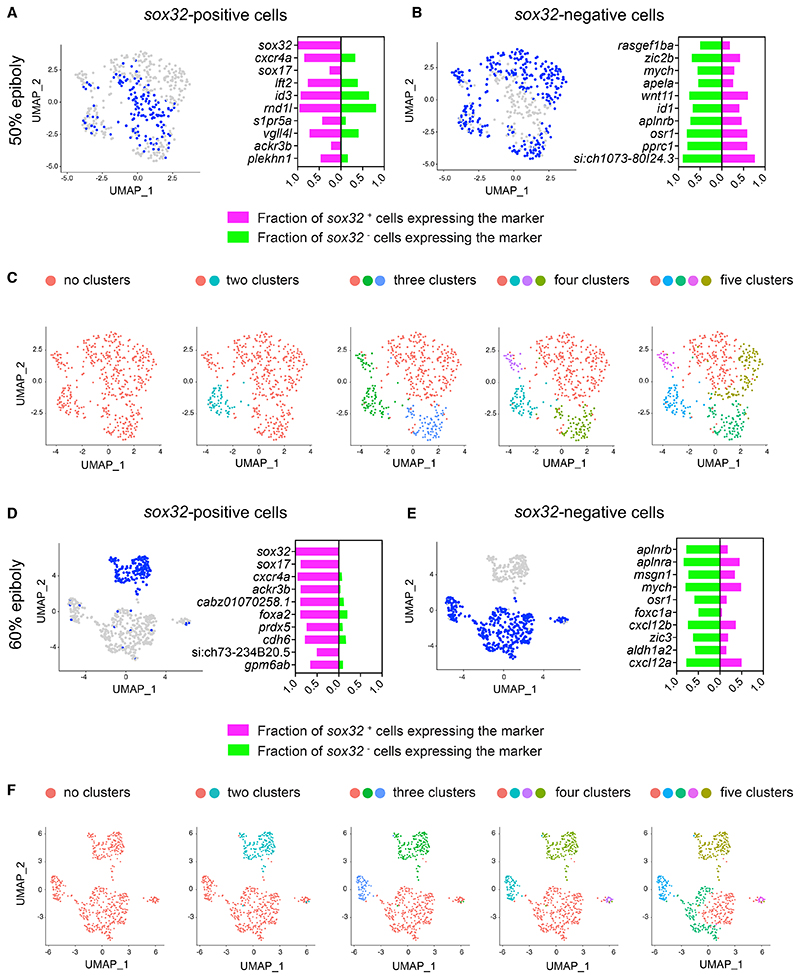
Switching to an endodermal fate is not associated with suppression of mesodermal fate (A) Left: uniform manifold approximation and projection (UMAP) visualization of *gata5*-positive cells derived from 50% epiboly stage embryos. Blue cells are those expressing at least one read count for *sox32*. Right: stacked bar plot showing the pct (percentage of expressing cells) for the top 10 genes differentially expressed in *sox32*-positive cells (magenta) compared to the pct for the same genes in *sox32*-negative cells (green). (B) Left: as in (A), but blue cells are those expressing less than one read count for *sox32*. Right: stacked bar plot showing the pct for the top 10 genes differentially expressed in *sox32*-negative cells (green) compared to the pct for the same genes in *sox32*-positive cells (magenta). (C) UMAP visualization of *gata5*-positive cells at 50% epiboly. Cells were clustered with increasing granularity across five iterations, Find cluster resolution = 0.1 to 0.5. Color coding refers to the different clusters. (D) As in (A), but for 60% epiboly embryos. (E) As in (B), but for 60% epiboly embryos. (F) As in (C), but for 60% epiboly embryos, Find cluster resolution = 0.01 to 0.5. See also Figures S5 and S6.

Switching to an endodermal fate is not initially associated with suppression of mesodermal markers So far, our data indicate that within the first two cell tiers of the margin, bipotential progenitor cells expressing early mesodermal markers can randomly switch to the endodermal fate. We next investigated whether this switch also involved the suppression of their mesodermal character, and reciprocally whether we could find evidence for a unique mesodermal master regulator. To capture transcriptional changes underlying endoderm and mesoderm specification, we used the same published scRNA-seq dataset^32^ used above for the cell cycle analysis to investigate gene expression at the embryo margin. To narrow down the analysis to the first few cell tiers, we focused on *gata5*-positive cells at 50% epiboly (5.3 hpf), which display mesodermal and endodermal marker expression and are devoid of more anterior ectodermal inputs (Figures S2C, S2D, S5A, and S5B). We performed differential gene expression analysis on cells positive or negative for *sox32* within the *gata5*-positive pool (Figures 5A and 5B; Data S2). Surprisingly, at 50%, epiboly gene expression across the two populations was rather homogeneous. Indeed, beside *sox32*, genes highly represented in endodermal progenitors were also present in their mesodermal progenitor neighbours like *cxcr4a* and *id3* (Figure 5A). Furthermore, mesodermal markers like *aplnrb* and *wnt11* were symmetrically expressed in endodermal progenitors (Figure 5B). Thus, there was no evidence for expression of a mesodermal master regulator equivalent to Sox32 for the endoderm, and the mesodermal signature was not suppressed in the endoderm progenitors.

As a more global approach to assess transcriptional heterogeneity at the margin, we clustered *gata5*-positive cells into different numbers of transcriptionally defined populations to ask whether *sox32*-positive cells would define a distinct cluster (Figure 5C). Strikingly, *sox32*-positive cells distributed across multiple clusters and these cells displayed analogous levels of *sox32* counts irrespective of their cluster allocation (Figures 5C and S5E). These findings were confirmed by repeating this analysis on cells positive for *mixl1*, which is expressed in a domain of five cell tiers at the margin and defines a transcriptionally distinct cluster at 50% epiboly (Figures S2C, S2D, S6A–S6C, and Data S3). Together these data show that by 50% epiboly, a subset of progenitors at the margin switches on *sox32* within an otherwise transcriptionally homogeneous pool.

After initial induction of *sox32*, cells proceed towards the endodermal lineage and express *sox17*. We therefore asked whether *sox32*-positive cells would diverge from their mesodermal counterparts during gastrulation. We repeated the same transcriptional analysis at 60% epiboly, which corresponds to mid-gastrulation (Figures 5D–5F, S5C, S5D, S6D, S6E, Data S4, and Data S5). We found that, together with *sox32*, several endoderm-specific markers, such as *sox17* and *ackr3b*, were now robustly and uniquely expressed in the endodermal progenitors; this was accompanied by a sharper suppression of mesodermal markers like *msgn1* and *aplnrb* (Figures 5D and 5E). Consistent with these observations, at 60% epiboly, *sox32*-positive cells could be readily identified within a transcriptionally distinct cluster (Figures 5F, S5F, and S6F) that uniquely expressed high levels of *sox32*, showing that by this time they have acquired a distinct identity.

These finding were also confirmed quantitatively by measuring cell-to-cell transcriptional distances between *sox32*-positive and *sox32*-negative cells at the margin (Figures S6G and S6H). Therefore, the switching of cells to an endodermal fate from bipotential progenitors that would otherwise differentiate to mesoderm is initially dependent on just the onset of *sox32* expression, with the two cell fates only becoming transcriptionally distinct by mid-gastrulation.

### The role of Nodal and Fgf signaling in mesoderm induction

**Figure 6:**
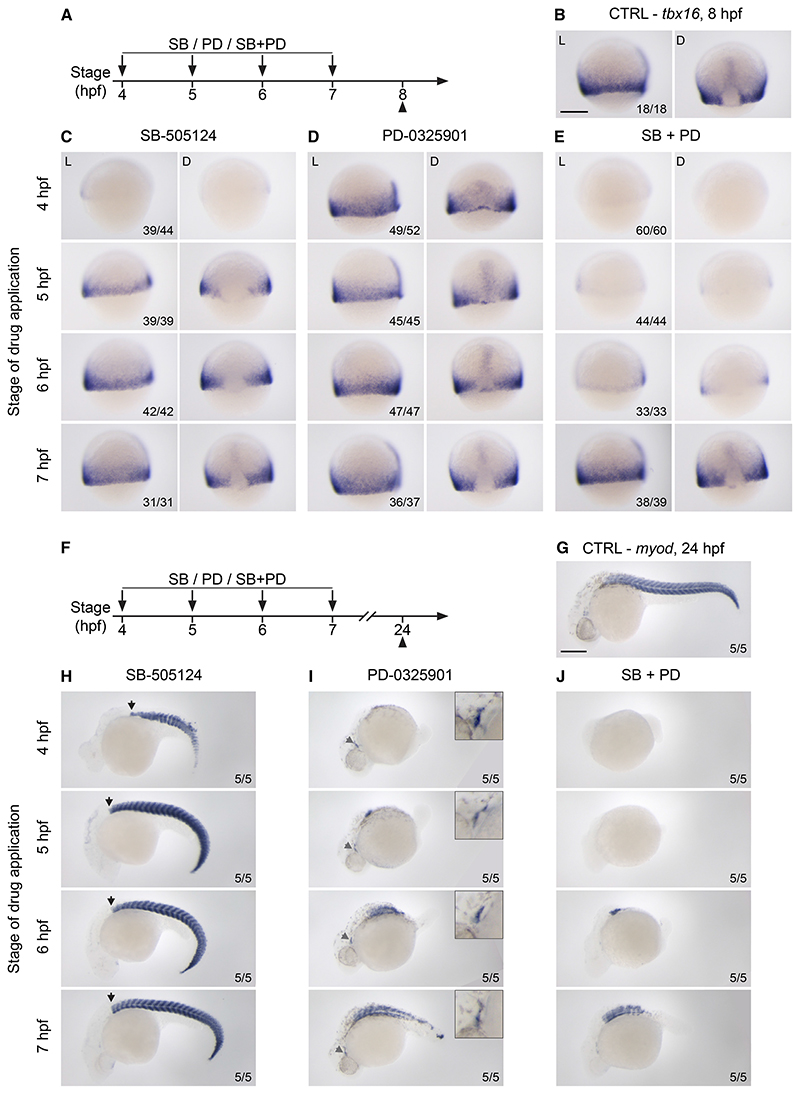
Nodal and Fgf signaling are required for mesoderm induction (A–E) WISH showing lateral (L) and dorsal views (D) of 8-hpf epiboly embryos stained for *tbx16*. Embryos underwent a time series of drug treatments with either 50 μM SB-505124 (left), 5 μM PD-0325901 (middle) or the combination of the two (right). Drug treatments were started at either 4 hpf, 5 hpf, 6 hpf, or 7 hpf, and embryos were fixed at 8 hpf epiboly (A). 8-hpf epiboly control (CTRL) embryos are shown for comparison (B). Note that for embryos treated from sphere stage with SB-505124 or the combination of SB-505124 and PD-0325901 the dorsal side cannot be unequivocally identified, as early Nodal inhibition prevents both *tbx16* expression and the formation of the dorsal shield. Therefore, for these samples, side views of representative embryos are shown. (F–J) As in (A–E), but embryos were fixed at 24 hpf and stained for *myod*. 24-hpf CTRL embryos shown for comparison (G). In this case, all views are lateral. In (H), the black arrow marks the most anterior position of the somites. In (I), the gray arrow indicates the jaw muscle (shown magnified, top right). Numbers in the bottom right of the images refer to the number of embryos showing the phenotype out of the total number studied. Scale bars: 250 μM (B-G).

Although we have shown that Nodal signaling is required for induction but not maintenance of endoderm progenitors, it is not clear whether the same is true for mesoderm progenitors. To understand the relative importance of the timing of Nodal and Fgf signaling for mesoderm induction, we performed timed inhibition of Nodal and Fgf/P-Erk signaling prior to and up to mid-gastrulation (4–7 hpf) (Figure 6A) and assessed the consequences for the maintenance of paraxial mesoderm progenitors (marked by *tbx16* at 8 hpf) (Figures 6B–6E) and their derivatives, such as somitic muscles in the trunk and tail and jaw muscles in the head (marked by *myod* at 24 hpf) (Figure 6G–6J).

Inhibition of Nodal signaling from 4 hpf suppressed expression of *tbx16* at the margin (Figure 6C). This early effect was followed by the loss of both head and trunk mesoderm at 24 hpf (although tail mesoderm, which is known to only partially depend on Nodal signaling, was maintained) (Figure 6H)^10,11^. In contrast, *tbx16* expression was maintained if Nodal inhibition occurred from 5 hpf onwards (Figure 6C), and expression of *myod* in the trunk was also restored under these conditions (Figure 6H). This suggested that, after 5 hpf, mesoderm derivatives can still form independently of Nodal signaling. In contrast to Nodal inhibition, in the absence of Fgf signaling at 4 hpf, *tbx16* was still expressed around the margin (Figure 6D). Also, while the early inhibition of Fgf signaling resulted in the expected loss of posterior paraxial derivatives in the embryo by 24 hpf (Figure 6I),^41^ these embryos displayed normal jaw muscles in the head (Figure 6I), indicating that in the absence of Fgf signaling, anterior paraxial mesoderm derivatives are still maintained. Strikingly, loss of both Nodal and Fgf signaling abolished *tbx16* expression when treatment was performed at 4, 5, or 6 hpf (Figure 6E) and resulted in the loss of almost all mesodermal derivatives at 24 hpf (Figure 6J). Therefore, even though in the absence of Nodal signaling the formation of paraxial mesoderm derivatives can be maintained by Fgf signaling, mesoderm specification does not occur in the absence of both Nodal and Fgf signaling.

Taken together, these data show that, unlike endoderm progenitors, specification of paraxial mesoderm progenitors relies on a sustained signaling input. This cannot be explained by Nodal signaling levels alone but requires a combination of both Nodal and Fgf signaling.

### Endoderm development is robust to variation in initial progenitor number

Our stochastic model for endoderm specification raises an interesting question: if the induction of endoderm is effectively a random process, how is the number of endodermal progenitors regulated? We hypothesized that zebrafish embryos might be robust to changes in the initial number of endodermal progenitors.

**Figure 7:**
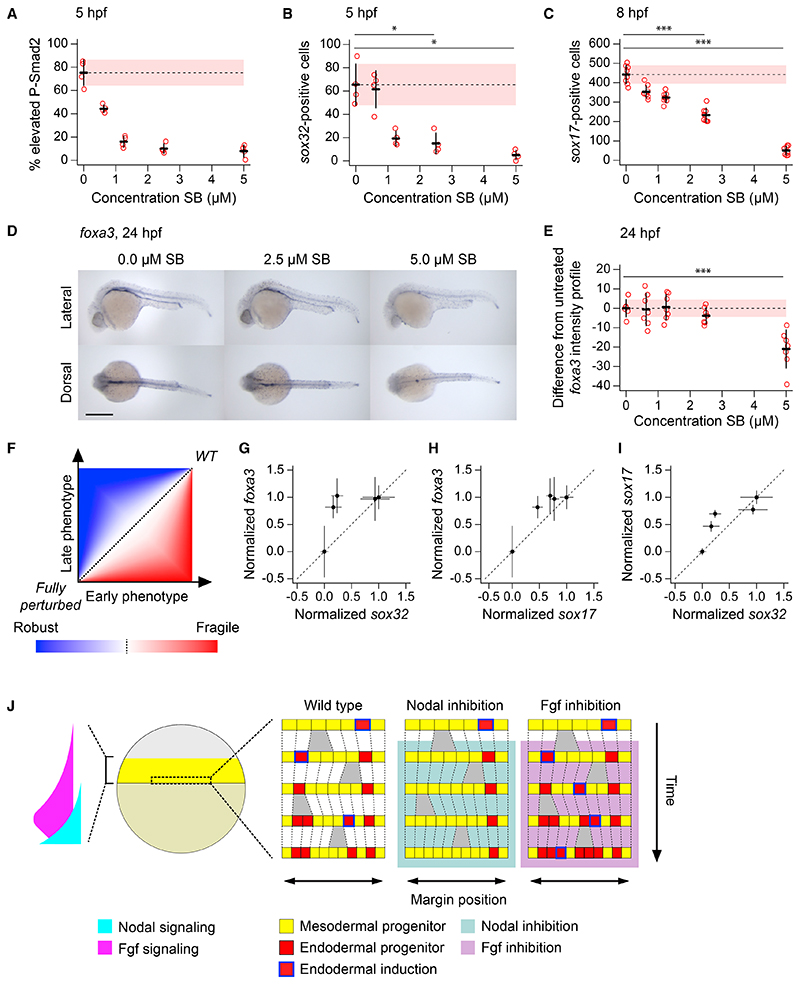
Endoderm induction is robust to variation in initial progenitor number (A) Plot showing percentage of cells in cell tiers 1 and 2 with elevated staining intensity for P-Smad2,for 5-hpf embryos treated at 4 hpf with different doses of SB-505124. Elevated P-Smad2 is defined by the mean background level for all embryos, with background for each embryo defined by the 99^th^ percentile in P-Smad2 staining intensity for cell tiers 9 and 10. Four embryos per dose. Means ± SD are shown. (B) Number of *sox32*-positive cells for the same embryos as in (A). Means ± SD are shown. (C) Number of *sox17*-positive cells for 8-hpf embryos treated at 4 hpf with different doses of SB-505124. Eight embryos per dose. Means ± SD are shown. (D) Representative 24-hpf embryos showing *foxa3* expression on treatment with different doses of SB-505124 in dorsal and lateral views. Anterior to left; in lateral view dorsal is up. (E) Quantification of the deviation of the *foxa3* staining intensity profile from the mean untreated, for 24-hpf embryos treated at 4 hpf with different doses of SB-505124. Means ± SD are shown. Note that in A-C and E, dashed line denotes untreated mean, with pink shaded area as one SD. (F) Schematic illustrating robustness and fragility in developmental systems, through plotting developmental states of embryos at early versus late time points under a range of perturbation from wild type (WT) to fully perturbed. The blue region, where the deviation from WT is greater at early stages under a given strength perturbation (relative to fully perturbed case), represents robustness, while the red area where the converse is true, represents fragility. (G–I) Comparison of early endoderm development with later measures using quantifications in (B, C, and E) to identify robustness in development as described in (F). Comparison of *sox32* counts at 5 hpf with *foxa3* expression profiles at 24 hpf (G). Comparison of *sox17* counts at 8 hpf with *foxa3* expression profiles at 24 hpf (H). Comparison of *sox32* counts at 5 hpf with *sox17* counts at 8 hpf (I). (J) Model of endoderm progenitor specification in early zebrafish development. The Nodal and Fgf gradients at the margin are schematized^3^. For details, see text. See also Figure S7. Scale bars: 250 μM (D). *p < 0.05; ***p < 0.001

To perturb endodermal progenitor number, we treated sphere-stage embryos with increasing doses of the Nodal receptor inhibitor SB-505124. Increasing inhibitor dose progressively reduced levels of Nodal signaling (Figure 7A) and resulted in a progressive reduction in endodermal progenitor number (Figure 7B). We saw this same effect later in epiboly upon counting the number of *sox17*-positive cells at 8 hpf (Figure 7C), or by assessing *sox17* transcript levels at 6 hpf (Figure S7G). However, it was already evident that numbers of endoderm progenitors were starting to be corrected by 8 hpf, as the graph of *sox17*-positive cells versus SB-505124 concentration is more linear than that of *sox32*-positive cells versus SB-505124 concentration (compare Figures 7B and 7C).

We next investigated how this early reduction in endodermal progenitors affected endoderm formation later in development. We maintained embryos until 24 hpf and visualized endodermal derivatives by in situ hybridization for *foxa3*^16^ (Figure 7D). To quantitatively determine how Nodal inhibition affected gut development, we measured the staining intensity along the gut and produced a mean intensity profile for untreated (DMSO) embryos (Figures S7A–S7F). Plotting the deviation from this untreated profile against Nodal inhibitor concentration revealed that there was initially no effect on the *foxa3* staining profile at lower doses of SB-505124 (Figures 7E and S7E). Only upon treatment with 5 μM SB-505124 is the *foxa3* staining profile significantly different from the untreated embryos (Figures 7E and S7F). Strikingly, the amount of endoderm at 24 hpf when embryos were treated with 2.5 μM SB-505124 was at wild-type levels, despite the number of progenitors being reduced to around 30% of the number in untreated embryos at 5 hpf and around 50% at 8 hpf (Figures 7B, 7C, and 7E). Similar results were obtained for embryos treated with 1.25 μM SB-505124. These results were confirmed by assaying *foxa3* transcript levels by qPCR at 24 and 48 hpf and comparing them to *sox17* expression at 6 hpf (Figures S7G, S7H–S7J, and S7M). The same trend was also observed for additional endodermal makers like *nkx2.3*, which marks pharyngeal arches and pharynx ^42^ (Figures S7K and S7M), and *pdx1*, which is a marker for liver and pancreas (Figures S7L and S7M).^16^.

This reduction in the early number of endodermal progenitors without an effect on the later phenotype demonstrates that endoderm development is robust to variation in early progenitor numbers (Figures 7F–7I), with some buffering even occurring during epiboly. Taken together, these results indicate that zebrafish embryos are robust to significant variation in the number of early endodermal progenitors, suggesting that to reproducibly produce a viable 24-hpf embryo, the amount of early induced endoderm need not be tightly regulated.

## Discussion

### A model for Nodal morphogen function

Here we describe a stochastic cell-fate-switching model for the role of Nodal signaling in the specification of the endoderm and mesoderm. This model not only differs from previous interpretations of how Nodal specifies these germ layers as a classical morphogen but also proposes a fundamentally different mode of action for morphogens in general (Figure 7J). Our data indicate that Nodal signaling provides competency for stochastic switching of biopotential progenitors to an endodermal fate, with low levels of Erk signaling favoring this switching.

In contrast to existing morphogen models, our present work also demonstrates that endoderm and mesoderm induction are essentially independent processes. On the one hand, as demonstrated here and in previous studies, Nodal works with and via Fgf to specify all cells in the margin of the embryo (up to 10 cell tiers) to a mesodermal fate.^21^ However, endoderm induction occurs in addition to this process. High levels of Nodal signaling in the cells closest to the margin confer a competency to switch to the endodermal fate. Cells that do not switch go on to differentiate to mesoderm. Switching is a stochastic process, where levels of Nodal and Fgf signaling determine the likelihood of a cell fate switch but do not determine cell fate *per se*. Increasing levels of Nodal signaling make the switch to endoderm fate more likely, and increasing levels of Fgf make it less likely (Figure 7J).

Consequently, in our model, *sox32* expression is not simply a readout of high levels (or an extended duration) of Nodal signaling, as would be the case in a classical morphogen model. Rather, *sox32* expression is regulated by a bistable switch, with Nodal signaling necessary (but not sufficient) for its transcriptional induction but not for its maintenance and associated commitment to the endodermal lineage. Indeed, Sox32 is known to induce its own transcription in conjunction with the homeobox transcription factor Pou5fl.^43^ Paraxial mesoderm fate is also maintained in the absence of Nodal signaling. However, while commitment of cells to the mesodermal fate can be lost by withdrawing both Nodal and Fgf signaling, this is not the case for endoderm, where the lineage is maintained despite removal of Nodal and Fgf signaling. Thus, while endodermal cells are specified through a transcriptional switch which renders them insensitive to the withdrawal of Nodal, mesodermal progenitors require a sustained signaling input (through Nodal or Fgf).

### Nodal defines a window of competency

In our model, the switch to an endodermal fate is a probabilistic event in which at any time all cells close to margin have the potential to switch fate (with the likelihood of switching dependent on their signaling profile). Therefore, the number of endodermal progenitors that accumulate during early epiboly is a function of the duration of exposure to Nodal: the longer the exposure, the more cells will randomly switch fate. Thus, rather than determining cell fate, Nodal defines a window of competency during which stochastic switching in cell fate can occur.

This is not the first time that the duration of exposure to Nodal has been implicated in this cell fate decision. As stated above, it was previously proposed that the decision between endoderm and mesoderm (and, within mesoderm, different types of mesoderm) depended on the duration of exposure to Nodal.^44^ In particular, as the gradient of Nodal signaling grows from the margin of the embryo, only cells close to the margin that have experienced a long duration of signaling can be fated as endoderm.^3^ Our proposed role for the length of exposure to Nodal is fundamentally different. At any point during early epiboly between 5.0 and 5.5 hpf (late blastula to early gastrula stages), effectively any marginal cell can be induced to the endodermal fate, and all endodermal progenitors at this time have an equal endodermal potential (regardless of when they were induced). In support of this model, our scRNA-seq analysis revealed that the only endodermal marker identified during early epiboly was *sox32*. Transcriptionally, there was no other signature that distinguished endodermal progenitors from their mesodermal counterparts at these early stages. Therefore, the specification of the endoderm occurs through a transcriptionally homogeneous population undergoing a transcriptional (and morphogenetic) bifurcation, with the direction that a cell takes being solely determined by whether it has by chance switched on *sox32* expression during the competency window.

Stochasticity and robustness in early development A major feature of our model is the importance of transcriptional and signaling variability between cells in a particular spatial location within the embryo. For example, we have demonstrated that there is considerable variation between cells within the first two cell tiers in their levels of nuclear P-Smad2, and that this variation plays a role in determining which cells are most likely to switch to an endoderm fate. It should be noted that this role for levels of Nodal signaling is very different from the role for signaling levels in classical morphogen gradient models for germ layer separation. In these models, differences in Nodal signaling levels between cells are predictable and spatially structured (a gradient from the margin of the embryo) and therefore act as a source of positional information. The variation we report is spatially unstructured and therefore cannot impart positional information.

The importance of stochasticity or noise in development has become increasingly apparent in recent years.^45–47^ Many computational developmental models focus on how this inherent noisiness is a feature of developmental systems that needs to be buffered out to produce a precise pattern—for example, by the wiring of transcriptional circuits.^48,49^ In contrast, while variation and noise between cells plays an important part in our model, it is also a requirement for the patterning process. Induction of *sox32* and the endodermal lineage is a chance event. Only by allowing this to occur over hundreds of cells for an extended duration are a substantial number of endodermal progenitors induced. Without stochas-ticity, no endoderm would be induced.

Our model also contrasts with such buffered developmental models in that there is no requirement for a precise pattern to be initially generated. We have demonstrated that later development is robust, as the number of *sox32*-positive endodermal progenitors can be reduced to as little as one third of the number seen in untreated embryos with little effect on the later endodermal phenotype. This robustness is also seen in other aspects of zebrafish development. For example, zebrafish embryos recover from early defects in the development of the embryonic shield in *ndr1* mutants, which are null for one of the two early expressed Nodal ligands.^50^ Moreover, several recent BMP signaling studies have shown that either insufficient or excessive BMP signaling early on in development is compensated for later and therefore does not result in the dorsal-ventral patterning defects that would be expected.^51,52^ If variation in early development can be buffered out, there is no selective pressure to evolve a precise mechanism. Therefore, if robustness is a general feature of later development, we should perhaps expect to find more inherently stochastic and variable mechanisms across early development of the sort we describe here.

### Limitations of study

In this study, we correlate signaling levels for Nodal and Fgf at the margin with expression of endodermal and mesodermal markers. This was performed on fixed material through quantitation of intensity values within individual nuclei at both the margin and the broader embryo. To capture temporal changes in signaling dynamics, we sampled embryos every 15–30 min between 4 to 5.5 hpf. This was largely sufficient for capturing temporal variation in Nodal signaling levels through P-Smad2, which is known to be integrated over time by cells at the margin.^3,9,21^ In contrast to Nodal, P-Erk is known to stochastically fluctuate in time and as cells divide.^53–56^ Even though endodermal cells are specified within a broad spectrum of P-Erk levels (fitting with our non-deterministic model of endoderm induction), it is possible that, at least in part, the variability in which cells are induced to endoderm could be explained by oscillatory dynamics of Erk signaling, which can be addressed in the future through the use of live reporters, enabling faster temporal sampling as well as tracing individual progenitors through time.

## Star★Methods

Detailed methods are provided in the online version of this paper and include the following:

- KEY RESOURCES TABLE
- RESOURCE AVAILABILITY
  ∘ Lead Contact
  ∘ Materials Availability
  ∘ Data and Code Availability
- EXPERIMENTAL MODEL AND SUBJECT DETAILS
  ∘ Fish lines and maintenance
- METHOD DETAILS
  ∘ FISH and immunofluorescence
  ∘ WISH
  ∘ Drug treatments
  ∘ Image analysis
  ∘ Identifying and positioning sox32-positive endodermal progenitors
  ∘ Identification of mitotic endodermal progenitors through expression of P-H3
  ∘ Simulation
  ∘ Sc-RNAseq analysis
  ∘ RNA extraction, cDNA preparation and qPCR
- QUANTIFICATION AND STATISTICAL ANALYSIS
  ∘ Statistical analysis

## Star★Methods

### Key Resources Table

**Table:** 

Reagent Or Resource	Source	Identifier
Antibodies		
Anti-phospho-Smad2 (IF, Dilution: 1 in 500)	Cell Signaling Technology	Cat# 8828; RRID: AB_2631089
Anti-phospho-Erk (IF, Dilution: 1:500)	Sigma	Cat# M8159; RRID: AB_477245
Anti-Phospho-Histone H3 (IF, Dilution 1:500)	Cell Signaling Technology	Cat# 9706; RRID:AB_331748
anti-Dig-AP (WISH, Dilution: 1 in 5000)	Roche	Cat# 11093274910; RRID:AB_2313640
HRP-conjugated anti-mouse secondary antibody (IF, Dilution: 1:500)	Dako	Cat# P0447 RRID: AB_2617137
HRP-conjugated anti-rabbit secondary antibody (IF, Dilution: 1:500)	Dako	Cat# P0448 RRID: AB_2617138
Chemicals, peptides, and recombinant proteins		
Tyramide hydrochloride	Sigma	Cat# T2879
NHS-Fluorescein ester	ThermoFisher Scientific	Cat# 46410
Cy3 mono NHS ester	Sigma	Cat# PA13101
Cy5 mono NHS ester	Sigma	Cat# PA15101
Digoxigenin (Dig)-11-UTP	Roche	Cat# 11209256910
NBT/BCIP	Sigma	Cat# B5655
SB-505124	Tocris	Cat# 3263
PD-0325901	Merck	Cat# 444968
Critical commercial assays		
Multiplex Fluorescent Assay v2	ACDBio	acdbio.com
Deposited data		
Code used for image analysis	This paper^5^	https://doi.org/10.5281/zenodo.7286310
Experimental models: Organisms/strains		
Zebrafish *Danio rerio*: WT	N/A	N/A
Oligonucleotides		
*sox17* qPCR primer forward GCATCCGAAGGCCAATGAAC	This paper	N/A
*sox17* qPCR primer reverse GCTTTCCATGACTTACCAAGC	This paper	N/A
*nkx2.3* qPCR primer forward GGACCACGAAACGAAGAGCTG	This paper	N/A
*nkx2.3* qPCR primer reverse GCTGCTGCTTGAAGCGCC	This paper	N/A
*foxa3* qPCR primer forward CATCGCAAGCTCCAAATCT	This paper	N/A
*foxa3* qPCR primer reverse TGCAGATCCAGATGGTGCAT	This paper	N/A
*pdx1* qPCR primer forward CAGTGGACAGGCCCTTATATGGTC	This paper	N/A
*pdx1* qPCR primer reverse GATGTGTCTCTCGGTGAGGC	This paper	N/A
*actin* qPCR primer forward CGAGCTGTCTTCCCATCCA	This paper	N/A
*actin* qPCR primer reverse TCACCAACGTAGCTGTCTTTCTG	This paper	N/A
Software and algorithms		
FIJI (ImageJ)	Schneider et al.^65^	https://imagej.net/Fiji/Downloads
R computing	The R Foundation	https://www.r-project.org/
Other		
Antisense RNA probe *tbx16*: linearize EcoRi: polymerase T7	Used previously by Osborn et al.^66^	N/A
Antisense RNA probe *myod*: linearize Xbal: polymerase T7	Used previously by Weinberg et al.^67^	N/A
Antisense RNA probe *sox17*: linearize Ncol: Polymerase Sp6	Used previously by Alexander and Stainier^39^	N/A
Antisense RNA probe *foxa3*: linearize Apal: polymerase T3	Used previously by Field et al.^68^	N/A
Dr *tbxta*-Ci	ACDbio	REF: 483511
Dr *sox32*-C4	ACDbio	REF: 524941-C4
Dr *mixll* -C2	ACDbio	REF: 850191-C2
Dr*gata5*-C4	ACDbio	REF: 850201-C4
Dr *tbx16*-C3	ACDbio	REF: 833541-C3

### Resource Availability

#### Lead Contact

Further information and requests for resources and reagents should be directed to and will be fulfilled by the lead contact, Caroline Hill (caroline.hill@crick.ac.uk).

#### Materials Availability

This paper does not report the generation of any new unique reagents.

### Experimental Model and Subject Details

#### Fish lines and maintenance

Zebrafish (*Danio rerio*) were housed in 28°C water (pH 7.5 and conductivity 500 mS) with a 15 h on/9 h off light cycle. All zebrafish husbandry was performed under standard conditions according to institutional (Francis Crick Institute) and national (UK) ethical and animal welfare regulations. All regulated procedures were carried out in accordance with UK Home Office regulations under project license PP6038402, which underwent full ethical review and approval by the Francis Crick Institute’s Animal Ethics Committee. For time courses embryos were maintained at 28°C and collected at regular intervals.

### Method Details

#### FISH and immunofluorescence

RNAscope® *in situ* hybridization^58^ was performed using the RNAscope Multiplex Fluorescent v2 system, as previously described^59,60^ with a few modifications. Briefly, embryos were first incubated in 2% H_2_O_2_ to inactivate endogenous peroxidases, before rehydration into PBS + 0.1% Tween-20 (PBTw), followed by hybridization overnight with specified probes at 40°C. After extensive washing in PBTw and postfixing for 10 min in 4% PFA, embryos were successively incubated with reagents Amp1-3 for 20 min at 40°C, with each amplification followed by washing with 0.2 x saline/sodium citrate buffer + 0.01% Tween-20 (SSCTw). The different probes were then visualized successively, through incubation with the HRP reagent in the appropriate channel (e.g. HRP-C1) for 20 min at 40°C. This was then followed by extensive washing in SSCTw and then PBTw. HRP was detected by incubating embryos with tyramide (Sigma, #T2879) coupled to either fluorescein-NHS ester (Thermo Scientific, #46410), Cy3 mono NHS ester (Sigma, #PA13101) or Cy5 mono NHS ester (Sigma, #PA15101) in PBTw for 25 min in the dark. Following the addition of 0.001% H_2_O_2_, the signal was allowed to develop for 30 min, after which the HRP was inactivated by incubating for 1 h in 3% H_2_O_2_ in PBTw to allow the detection of the next probe by repeating the process for each channel.

Immunofluorescence for P-Smad2 and P-Erk was performed as described^21^ with minor modifications. Embryos were first incubated in 2% H_2_O_2_ to inactivate endogenous peroxidases, before rehydration into PBS/1% Triton-X (PBTr). After incubating in acetone at -20°C, embryos were blocked in PBTr + 10% fetal bovine serum (FBS), before incubating with antibodies against P-Smad2 (Cell Signaling Technology, #8828, 1:500), against P-Erk (Sigma, #M8159, 1:500) or against P-H3 (Cell Signaling Technology, #9706S, 1:500) at 4°C overnight. For visualization, embryos were incubated for 3 h at room temperature with HRP-conjugated anti-rabbit secondary antibodies for pSmad2 (Dako, #P0448, 1:500) or anti-mouse secondary antibodies for P-Erk and P-H3 (Dako, #P0447, 1:500), and visualized with the tyramide system (as describe above for RNAscope assays) to increase the sensitivity of signal detection.

When performing immunofluorescence following in situ hybridizations, HRP inactivation with 3% H_2_O_2_ was followed by extensive washing in PBTr and incubation in acetone at -20°C. After that, embryos were incubated for 2 h with PBTr + 10% FBS prior to incubation with antibodies against P-Smad2 or P-Erk and were processed as for conventional immunofluorescence and RNAscope assays. For visualization, embryos were incubated for 3 h at room temperature with HRP-conjugated secondary antibodies, and visualized with the tyramide system.

For both *in situ* hybridization and immunofluorescence, embryos were counter stained with DAPI to visualize nuclei. Embryos were then removed from the yolk, cut to allow flat-mounting, and mounted in glycerol before imaging the entire embryo on a Leica SP8 inverted confocal microscope, with an HC PL APO CS2 20x/0.75 IMM objective with the correction collar set for a glycerol immersion fluid.

### Wish

All plasmids for the generation of riboprobes, with references, can be found in the Key Resources Table. Standard WISH was performed as previously described.^20,21^ Briefly, samples were initially rehydrated to PBS/0.1% Tween (PTW). Next embryos were incubated with digoxigenin (Dig)-11-UTP-(Roche, #11209256910) labeled riboprobes in a hybridization mix containing 5% dextran sulphate overnight at 65°C. Embryos were then incubated at 4°C with anti-Dig-AP antibody overnight (Roche, #11093274910; 1:5000). After the incubation they were washed extensively in PTW before detecting alkaline phosphatase with NBT/BCIP (Sigma, # B5655).

#### Drug treatments

For drug treatments, the inhibitors PD-0325901 and SB-505124 were dissolved in DMSO and directly diluted in embryo medium at 5 μM (PD-0325901) and 10 or 50 μM (SB-505124) respectively, oras indicated in the Figures. Embryos were maintained at 28°C; time of treatment onset and durations are specified in the Figure legends.

#### Image analysis

Flat mounted embryos were imaged on a Leica SP8 confocal microscopy as above. Complete embryos were captured using a tile scanning, with the voxel size set to 0.125 x 0.125 x 0.250 μM. Images were captured in 16-bit depth.

To quantify nuclear staining intensities for all cells in an embryo, nuclei were segmented using a combination of packages from the FIJI image analysis software. To identify which voxels belonged to which nuclei compared to background, a local adaptive thresholding was run on each Z-slice. This was repeated a further twice, but after reslicing the Z-stack along the X- and Y-axes, and the intersection of the foreground voxels in the three thresholded stacks was taken as nuclear voxels. As this approach did not fully separate individual nuclei, a 3-dimensional watershed was performed on the unthresholded DAPI channel (following a Gaussian blur) using the Classic Watershed function in the MorphoLibJ package. The boundaries from the water-shedding were then used to separate the thresholded nuclei. The majority of background voxels picked up by the auto-thresholding were removed by a round of erosion and dilation.

Nuclei were identified as blocks of contiguous voxels using the 3D ROI Manager from the 3D ImageJ Suite in FIJI and were used as masks to measure the mean staining intensity for all nuclei in all channels of the same image stack. Finally, background objects were excluded through a further round of filtering: objects with a mean DAPI staining intensity below 1000 were removed, as were overly flat objects (which empirically mapped to background objects) identified as those where 1.25 * volume < surface area.

To allocate a marginal position for each point, the boundary between the embryo and the YSL was marked by hand on a maximum XY projection of the Z-stack. Fitting a spline curve gave an array of regularly spaced points (at pixel increments) around the margin of the embryo. By identifying dorsal –through the location of the DFCs, or by the dorsal domain of *tbxta* – each point was allocated a marginal position (for 0 to 360 degrees where 0 degrees is dorsal). The closest marginal point was identified for each embryonic nucleus, and the associated marginal position was assigned.

#### Identifying and positioning sox32-positive endodermal progenitors

Cells positive for *sox32* were identified relative to a staining intensity threshold. This was determined by inspection, using a value where cells clearly expressing *sox32* were recovered but background staining nuclei were excluded. As embryos within each dataset were stained together and imaged under the same settings, intensity values were comparable within a dataset. However, as different datasets were stained and imaged independently, the intensity values are not directly comparable, and so the threshold for identifying *sox32*-positive cells was identified independently for each dataset. All *sox32*-positive cells were allocated a position, 4, relative to the margin as described above.

Cells lying beyond the margin were removed as belonging to the YSL. Cells within 5 μM of the lowest nucleus in Z locally were also excluded as belonging to the YSL. DFCs were removed by excluding *sox32*-positive cells lying in the top 20% of the embryo (see Figure 2D; Figure S2G). To maintain a uniform circular distribution of *sox32*-positive cells upon removal of DFCs, the position of the remaining cells was scaled, giving a rescaled position, q. Recording marginal positions from -180 to 180 degrees (where 0 degrees is dorsal), for cells where 4 >0,w = 180-1.25*(180-4), and where 4 <0,w = -180-1.25*(-180-4).

#### Identification of mitotic endodermal progenitors through expression of P-H3

To determine the ratio of mitotic endodermal cells to other mitotic cells, embryos were fixed at 50% epiboly and stained for *tbx16, sox32* and the mitotic marker P-H3. They were flat mounted and imaged on a Leica SP8 confocal microscope at the same resolution as above. Initially, cell nuclei were segmented using the Cellpose^61^ plugin for Trackmate.^62^ The segmentation was performed in 2D using the Cytoplasm pretrained model with an 8-pixel estimated cell diameter. The 2D segmented images were then transformed into a 3D object labeled image by tracking individual slices through the Z-stack and integrating the 2D tracked objects into 3D objects.^63^ Intensities were measured using CellProfiler.^64^ The 3D object labeled image was transformed into individual 3D CellProfiler objects using the ‘Convert Image To Objects’ module. The integrated intensity enclosed by each object was then measured for each channel (DAPI, P-H3, *sox32* and *tbx16*). Endodermal cells expressing P-H3 were identified relative to a staining intensity threshold which was validated by visual inspection. For *tbx16* and P-H3, intensity thresholds were defined using mean nuclear intensity values. For *sox32*, mean nuclear intensity values were used in combination with the standard deviation of nuclear intensity. This enabled the more precise separation of *sox32*-positive nuclei from background given the spotty nuclear pattern of *sox32* in the RNAscope staining.

#### Simulation

The margin of the embryo was simulated as an array of cells dividing through time. Each run of the simulation was initiated with 500 cells, proliferating to 1000 by the end of the simulation. These numbers were based on empirical counts from the first two cell tiers of the segmented embryos (where endodermal progenitors are induced) across the early epiboly period when endoderm is induced. For simplicity it was assumed that the number of cells increases linearly through time, with each cell dividing once. Therefore, the simulation was divided into 500 time steps, with each cell in the array given an arbitrary, unique rank from 1 to 500, determining the time step each cell divides (with no cell or its daughters dividing twice during the simulation). At each time point, all cells in the array (regardless of whether or not they had yet divided) could turn on *sox32* expression, with a probability of 0.00015 (this probability was manually tuned to match the number of *sox32*-positive cells through time to the empirical data (Figure 2E)). Once induced, *sox32* expression was maintained through all remaining time steps (and in both daughters if the cell then divided). To allocate a marginal position to each cell, the array of cells was wrapped around a circle, with the two ends at 0 degrees, and all cells equally spaced.

#### Sc-RNAseq analysis

The URD zebrafish cell atlas^32^ was read into R using the URD package and processed using the Seurat R toolkit https://satijalab.org/seurat/articles/get_started.html (version 4.0.5). The URD R object was initially transformed into a Seurat object for downstream analysis. To investigate gene expression dynamics at the onset of gastrulation to mid-gastrulation, cells from 50% and 60% epiboly stages were initially isolated from the dataset and the raw count data were processed using Seurat’s standard data processing pipeline. For the selected stages, cells were classified into clusters (Find Cluster resolution = 0.08 for 50% epiboly and 0.05 for 60% epiboly) and UMAPs were generated using 15PCs for 50% epiboly and 20PCs for 60% epiboly. For most of the analyses in the study, cells at the margin were isolated by sub-setting cells that had at least one read of *gata5*. Alternatively, cells at the margin were obtained by isolating transcriptionally distinct clusters overlapping with the expression of *mixl1* (cluster 2 for 50% epiboly and cluster 1 and 2 for 60% epiboly). In both cases, cells were re-processed and displayed as UMAPs using 15PCs.

For cell cycle analysis, cell cycle scores were calculated for *gata5*-positive cells using the CellCycleScoring function in Seurat and cell cycle heterogeneity was visualized by performing PCA on cell cycle markers. Next, the number of cells in G1, G2/M or S phase in *sox32*-positive and -negative cells was extracted and normalized to total cell number for each population. For the analysis of *sox32* expression levels across the cell cycle, raw counts for *sox32* were extracted from *sox32*-positive cells in S phase, G2/M phase or G1 phase and plotted in GraphPad Prism 8. For gene enrichment analysis, the ‘find markers’ function (min.diff.pct = 0.1) was used on cells positive or negative for *sox32* within the *gata5*-positive pool. After that, the ptc.1 and ptc.2 values were extracted from each gene set and the values for the top 10 genes for each population were plotted in GraphPad Prism 8. Finally, for clustering analysis of *gata5*-positive cells at the margin, five clustering iterations were performed using the FindNeighbors and FindClusters functions (Find cluster resolution = 0.1 to 0.5 for 50% epiboly, 0.01 to 0.5 for 60% epiboly). The same analysis was also performed on cluster-derived marginal cells for both 50% and 60% epiboly stages (Find cluster resolution = 0.05 to 0.56 for 50% epiboly, 0.01 to 0.2 for 60% epiboly). See Data S1–S5 for full details of these analyses. To construct the distance matrix shown in Figure S6G and H, cluster-derived marginal cells at 50% and 60% epiboly were initially isolated. Then, for each sample a matrix containing cell barcodes (rows) and the 2000 most variable features (columns) was exported from Seurat for further analysis. The matrix was centered and used to run PCA using the prcomp function in the factoextra package (http://www.sthda.com/english/wiki/factoextra-r-package-easy-multivariate-data-analyses-and-elegant-visualization). The coordinates for PC1, PC2 and PC3 were then selected and scaled before generating the distance matrix using the ‘get_dist’ function followed by visualization using the ‘fviz_dist’ function also in the factoextra package. After removing null and duplicated values, cell-to-cell distances were sorted according to whether a cell was positive or negative for the expression of *sox32*. The resulting distances between cell groups were plotted in GraphPad Prism 8 and visualized as scatter dot plots.

#### RNA extraction, cDNA preparation and qPCR

Total RNA was isolated from pools of 15 embryos using the RNeasy Mini kit (Qiagen, Cat. No. 74106) as per the manufacturer’s instructions. To ensure complete genomic DNA removal, RNA extracts were digested with DNase I (Qiagen, Cat. No. 79254). Next, cDNA was generated with the AffinityScript kit (Agilent) as previously described.^21^ For qPCR, the cDNA was diluted 1:10 and amplified using the PowerUp SYBR Green Master Mix (ThermoFisher Scientific) with 300 nM of each primer and 2 ml of diluted cDNA. Fluorescence acquisition was performed on a QuantStudio 12 Flex machine (ThermoFisher Scientific). Primers are listed in the Key Resources Table. Finally, quantification for relative gene expression was performed using the comparative Ct method and gene expression was normalized to *actin*. Changes in gene expression upon SB-505124 treatment were obtained by normalizing the values at the different doses of SB-505124 to the DMSO treatment for each biological replicate.

### Quantification and Statistical Analysis

#### Statistical analysis

For testing of *sox32*-positive cell distributions in Figure 2D a Watson’s Test for Circular Uniformity (test statistic = 3.39) was used. For the gene expression analysis across the phases of the cell cycle, raw counts levels were compared using a non-parametric Kruskal-Wallis test with Dunn’s multiple comparison correction (Figure 2G, and S2F). A Kruskal-Wallis test with Dunn’s multiple comparison correction was also used to test differences in the ratio of P-H3 positive cells (Figure 2J). Fluorescence intensity for P-Smad2 and P-Erk between cell tiers at different time points was compared using a t-test (Figure 3D–H, S3A and B). A t-test was also used to compare *foxa3* profiles (Figure 7E) and the fluorescence intensity for *tbx16* in YSL vs blastoderm cells (Figure S2A) as well as qPCR data in Figure S7. Finally, a Wilcoxon rank sum test was used to compare the percentage of high P-Smad2 cells (Figure 4J), percentage of *sox32*-positive cells (Figure 4K) and the number of *sox17*-positive cells (Figure 4N and 4O) upon PD-0325901 and SB-505124 treatments.

## Supplementary Material

Data S1

Data S2

Data S3

Data S4

Data S5

Supplementary Material

## Data Availability

There are no new datasets reported in this paper. Code used for image analysis is available on the DevSigLab GitHub account (see https://doi.org/10.5281/zenodo.7286310).^57^ The sc-RNAseq analyses are provided as R markdown files (Data S1–S5). Any additional information required to reanalyze the data reported in this paper is available from the lead contact upon request.

## Highlights

- Levels of Nodal and Fgf signaling do not determine endodermal or mesodermal fate
- Nodal signaling promotes bipotential progenitor switching to endoderm fate
- Switching is a random event, and its likelihood is modulated by Fgf signaling
- Imprecise endoderm specification is corrected later to ensure robust gut formation

## In brief

Economou and Guglielmi et al. show that in zebrafish embryos Nodal signaling levels do not determine endodermal or mesodermal fate. Instead, Nodal signaling establishes progenitor competency to stochastically switch towards endoderm, a process antagonized by Fgf signaling. This early stochasticity is nevertheless buffered during development, leading to robust gut formation.
