# Supplementary material for: Nodal signaling establishes a competency window for stochastic cell fate switching
Source: Dev Cell. Author manuscript; Available in PMC 2023 Oct 26. (PMC7615190; doi:10.1016/j.devcel.2022.11.008)
Supplement: Supplementary Material [file EMS189029-supplement-Supplementary_Material.pdf]

Developmental Cell, Volume *57*

Supplemental information

Nodal signaling establishes a competency window for stochastic cell fate switching

Andrew D. Economou, Luca Guglielmi, Philip East, and Caroline S. Hill

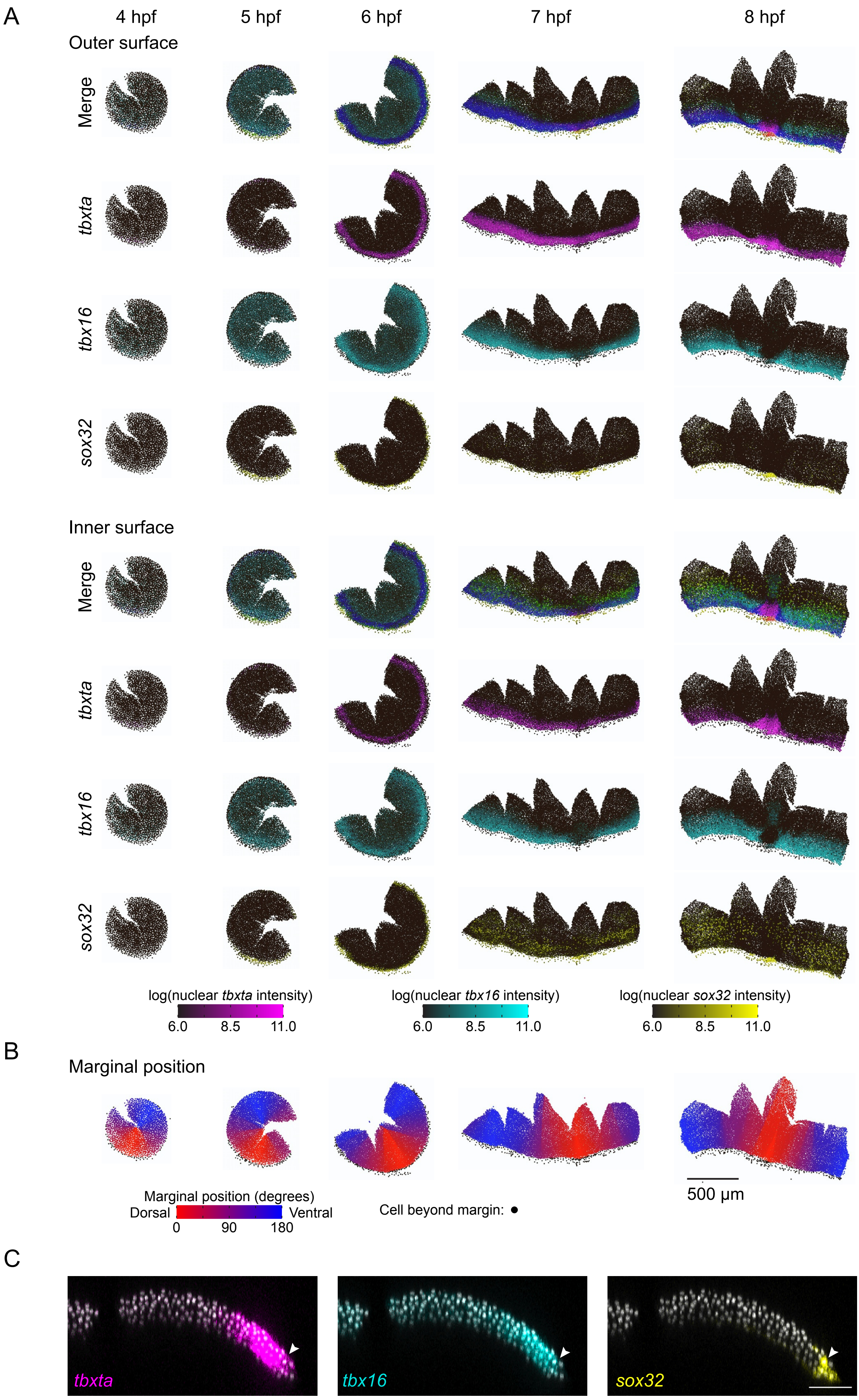
A
4 hpf
5 hpf
6 hpf
7 hpf
8 hpf
Outer surface
Merge
tbxta
tbx16
sox32
Inner surface
Merge
tbxta
tbx16
sox32
log(nuclear tbxta intensity)
6.0 8.5 11.0
log(nuclear tbx16 intensity)
6.0 8.5 11.0
log(nuclear sox32 intensity)
6.0 8.5 11.0
B
Marginal position
500 µm
Marginal position (degrees)
Dorsal
Ventral
0 90 180
Cell beyond margin: ●
C
tbxta
tbx16
sox32

Figure S1

Figure S1. Gene expression patterns are captured through nuclear segmentation.

(A) Time series of representative reconstructed embryos for each stage, showing gene expression patterns from nuclear segmentation (*tbxta*, magenta; *tbx16*, cyan; *sox32*, yellow). For each embryo, the XY positions of the centroids for each segmented nucleus are plotted, colored by the intensity of staining for each gene (nuclei below the background level of staining intensity 6.0 are colored black). The order in which nuclei are plotted is determined by their Z coordinate, giving a view of the outer surfaces of the embryos (upper panels), or by reversing this order, the inner surfaces (lower panels).

(B) Reconstructed embryos in (A) with each nucleus colored by the dorsoventral position of the closest point on the embryonic margin (dorsal, red; ventral, blue). Nuclei lying beyond the margin are colored black. Scale bar, 500 µm.

(C) Z-reconstruction from a 12.5-µm thick optical slice showing a 6-hpf embryo stained with RNAscope in situ hybridization for *tbxta*, *tbx16* and *sox32*. Arrowheads show a nucleus positive for all three markers. Scale bar, 100 µm.

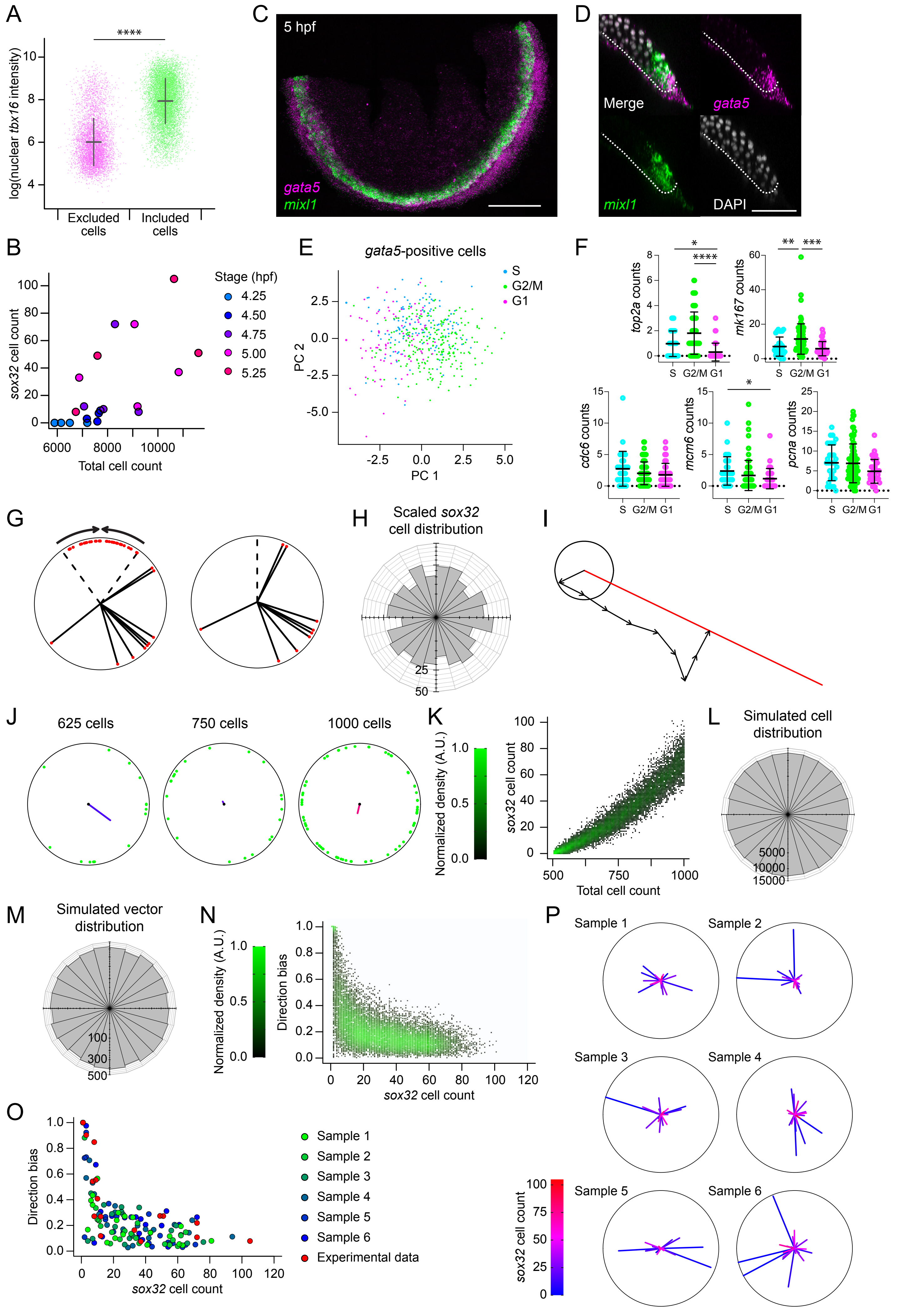

A
****
log(nuclear tbx16 intensity)
Excluded cells
Included cells
C
5 hpf
gata5
mixl1
D
Merge
gata5
mixl1
DAPI
B
sox32 cell count
Total cell count
Stage (hpf)
4.25
4.50
4.75
5.00
5.25
E
gata5-positive cells
S
G2/M
G1
PC 1
PC 2
F
top2a counts
mk167 counts
cdc6 counts
mcm6 counts
pcna counts
G
H
Scaled sox32 cell distribution
I
J
625 cells
750 cells
1000 cells
K
Normalized density (A.U.)
sox32 cell count
Total cell count
L
Simulated cell distribution
M
Simulated vector distribution
N
Normalized density (A.U.)
Direction bias
sox32 cell count
O
Direction bias
sox32 cell count
Sample 1
Sample 2
Sample 3
Sample 4
Sample 5
Sample 6
Experimental data
P
Sample 1
Sample 2
Sample 3
Sample 4
Sample 5
Sample 6
sox32 cell count

Figure S2

Figure S2. Endodermal progenitors are induced randomly in space and time.

(A) Plot of nuclear *tbx16* intensity for 20 embryos collected from mass spawning across early epiboly (same embryos as used in Figure 2D), for all nuclei up to the second cell tier (the distance from the margin is less than 30 µm – this includes cells beyond the margin where the distance is negative). Cells are separated by whether they lie within the margin and are further than 5 µm from the lowest local nucleus in Z (included) or otherwise not (excluded). The lack of overlap between the two populations shows that these criteria can be used to separate the *tbx16*-positive blastodermal cells from the *tbx16*-negative YSL. Means ± SD are shown. Statistical difference was tested using a t-test. $p < 0.0001$.

(B) Plot showing relationship between number of *sox32*-positive endodermal progenitors (YSL and DFCs removed) and total cell number for embryos collected at 15-min intervals from a mass spawning. Colors indicate embryonic stage of each point.

(C) Maximum projection of RNAscope in situ hybridization for *gata5* and *mixl1*, in a flat-mounted 5-hpf embryo. Scale bar, 250 µm.

(D) Z-reconstruction from a 12.5 µm thick optical slice through the lateral region of the embryo, showing restriction of *gata5* expression to the most marginal cells, with *mixl1* expressed in a slightly larger domain. Dashed line marks the boundary between the embryo and YSL. Scale bar, 100 µm.

(E) Principal Component Analysis (PCA) performed on *gata5*-positive cells using cell cycle markers. Color coding indicates the cell cycle phase: S phase (cyan), G2/M (green), G1 (magenta).

(F) Scatterplots showing expression levels of cell cycle markers in *sox32*-positive cells. Color coding as in (E). Means ± SD are shown. Dotted line indicates zero. Statistical difference was tested using a Kruskal-Wallis test with Dunn's multiple comparison correction. For *top2a*: $p < 0.0001$ (G2/M vs G1), $p < 0.05$ (G1 vs S), n.s (G2/M vs S). For *mk167*: $p < 0.001$ (G2/M vs G1), n.s (G1 vs S), $p < 0.01$ (G2/M vs S). For *mcm6*: n.s (G2/M vs G1), $p <0.05$ (G1 vs S), n.s (G2/M vs S). For *cdc6* and *pcna* no significance was found across comparisons.

(G) Cells positive for *sox32* in the top 20% of the embryo, corresponding to the dorsal side are excluded to remove DFCs (left panel). For *sox32*-positive progenitors in the ventrolateral region to conform to a uniform circular distribution, the angular positions are scaled (right panel).

(H) Distribution of 489 *sox32*-positive cells around the embryonic margin as in Figure 2D, scaled to account for the removal of DFCs. The distribution of cells could not be distinguished from a uniform circular distribution (Watson's Test for Circular Uniformity: test statistic = 0.0379, p-value > 0.10).

(I) To generate an average direction vector for each embryo, the individual vectors for each cell in an embryo were summed (black arrows). A direction bias was calculated by dividing the

total vector length (red line) by the length of the summed vectors, where the length of each individual vector is 1, and the total length is the number of endodermal progenitors.

(J) Examples of three simulated embryos at different time steps (from 500 cells to 1000 cells), with endodermal progenitors in green, showing direction vectors and directional bias.

(K) Plot showing the total number of *sox32*-positive endodermal progenitors through time (given by the total number of cells in the simulation). Based on a total of 10,000 simulations, with 100 embryos terminating at 100 equally spaced time points, ranging from 5 cell divisions to the full simulation (total cell number doubling from 500 to 1000).

(L) Spatial distribution of *sox32*-positive progenitor cells pooled from all 10,000 simulations. The spatial distribution of cells is indistinguishable from uniform (Watson's Test for Circular Uniformity: test statistic = 0.1175, p-value > 0.10).

(M) Spatial distribution of direction vectors from all 10,000 simulations. The spatial distribution of vectors is indistinguishable from uniform (Watson's Test for Circular Uniformity: test statistic = 0.0833, p-value > 0.10).

(N) Plot of directional bias against the total number of *sox32*-positive cells, across all 10,000 simulations.

(O) Comparison of six random subsamples of 20 simulated embryos distributed through time with the experimental data (in red). This shows the similarity in the relationship between the directional bias and the total number of *sox32*-positive cells between the experimental and simulated data.

(P) Plots of directional bias for the same six embryos as in (O), showing similarity between simulated data and experimental (see Figure 2K).

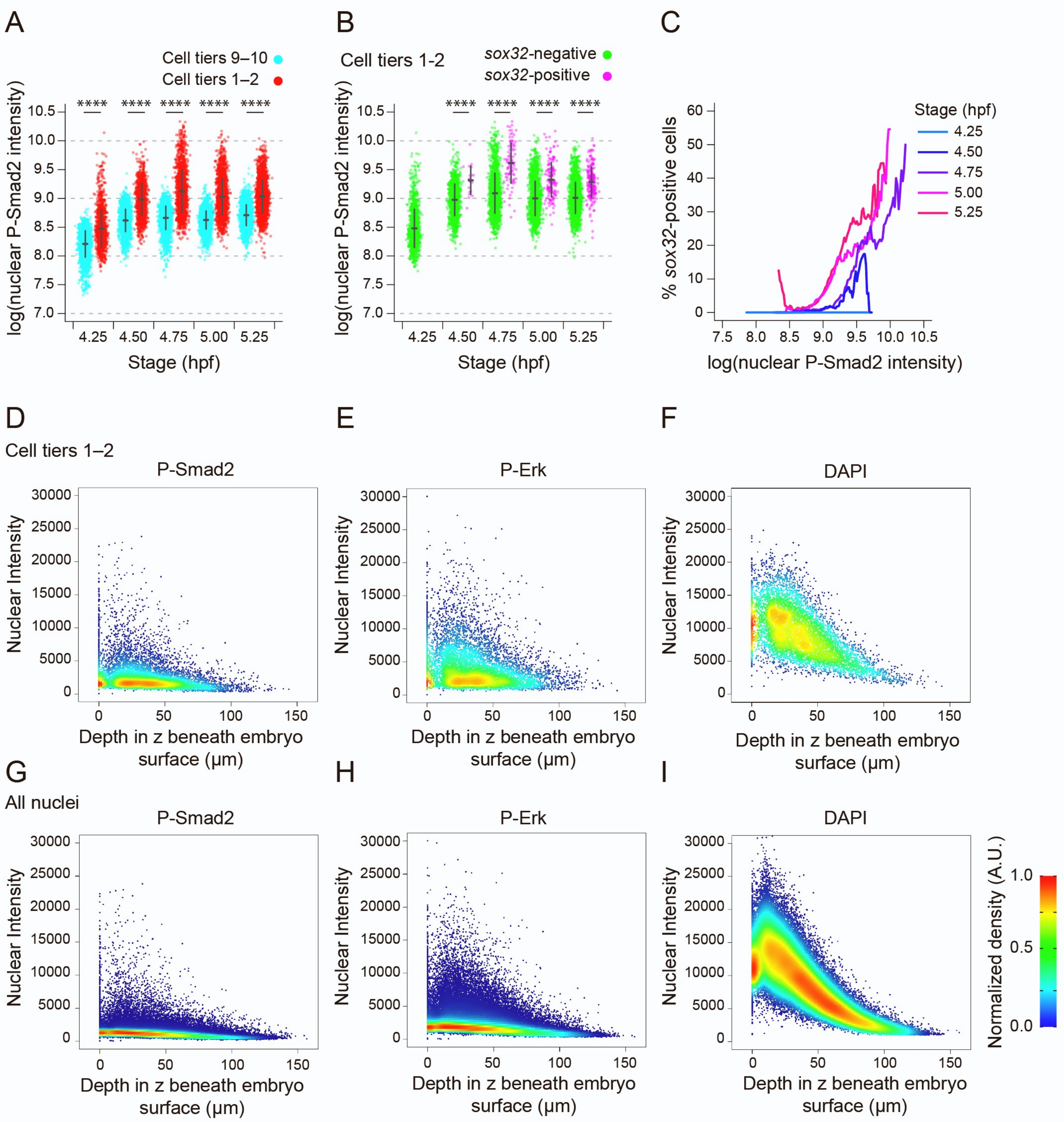
A
Cell tiers 9–10
Cell tiers 1–2
****
log(nuclear P-Smad2 intensity)
Stage (hpf)
B
Cell tiers 1-2
sox32-negative
sox32-positive
C
% sox32-positive cells
Stage (hpf)
4.25
4.50
4.75
5.00
5.25
log(nuclear P-Smad2 intensity)
D
Cell tiers 1–2
P-Smad2
E
P-Erk
F
DAPI
Nuclear Intensity
Depth in z beneath embryo surface (μm)
G
All nuclei
P-Smad2
H
P-Erk
I
DAPI
Normalized density (A.U.)

Figure S3

Figure S3. Nodal signaling levels are not deterministic for cell fate switching

(A) Plot showing P-Smad2 staining intensity for all cells in the first and second cell tiers compared to background levels (ninth and tenth cell tiers) for mass spawned dataset (Figure S2B). Means ± SD are shown. Statistical difference was tested using a t-test. For all timepoints: $p < 0.0001$.

(B) Plot showing P-Smad2 staining intensity for all cells in the first and second cell tiers, broken down into *sox32*-positive and *sox32*-negative cells. Means ± SD are shown. Statistical difference was tested using a t-test. For all timepoints beside 4.25 hpf: $p < 0.0001$.

(C) Traces showing the proportion of cells that are *sox32*-positive for a given P-Smad2 staining intensity for cells in the first and second cell tiers at different stages. The proportion of *sox32*-positive cells for a given level of P-Smad2 staining is based on all cells within a window of ± 10% the total range of P-Smad2 staining intensities for that stage (as this dataset was from a mass spawning and therefore more variable than the single clutch dataset, a larger smoothing window was used).

(D) Density scatter plots showing nuclear fluorescence intensity staining for P-Smad2 (y-axis) against the distance in Z of the nucleus from the surface of the embryo (x-axis). Plot shows cells belonging to the first two cell tiers of the embryo margin from the dataset shown in Figure 3.

(E) Density scatter plots showing nuclear fluorescence intensity staining for P-Erk (y-axis) against the distance in Z of the nucleus from the surface of the embryo (x-axis). Plot shows cells belonging to the first two cell tiers from the same embryos as in (D).

(F) Density scatter plots showing nuclear fluorescence intensity staining for DAPI (y-axis) against the distance in Z of the nucleus from the surface of the embryo (x-axis). Plot shows cells belonging to the first two cell tiers from the same embryos as in (D).

(G) As in (D), but all nuclei in the embryo are plotted

(H) As in (E), but all nuclei in the embryo are plotted

(I) As in (F), but all nuclei in the embryo are plotted. Note that, in contrast to the DAPI staining which shows a loss of signal with depth (indicated by the slope of the red labeling, which represents the background signal), for P-Smad2 and P-Erk the loss of signal due to depth is much less than the difference in signal due to real differences in expression (blue labeling).

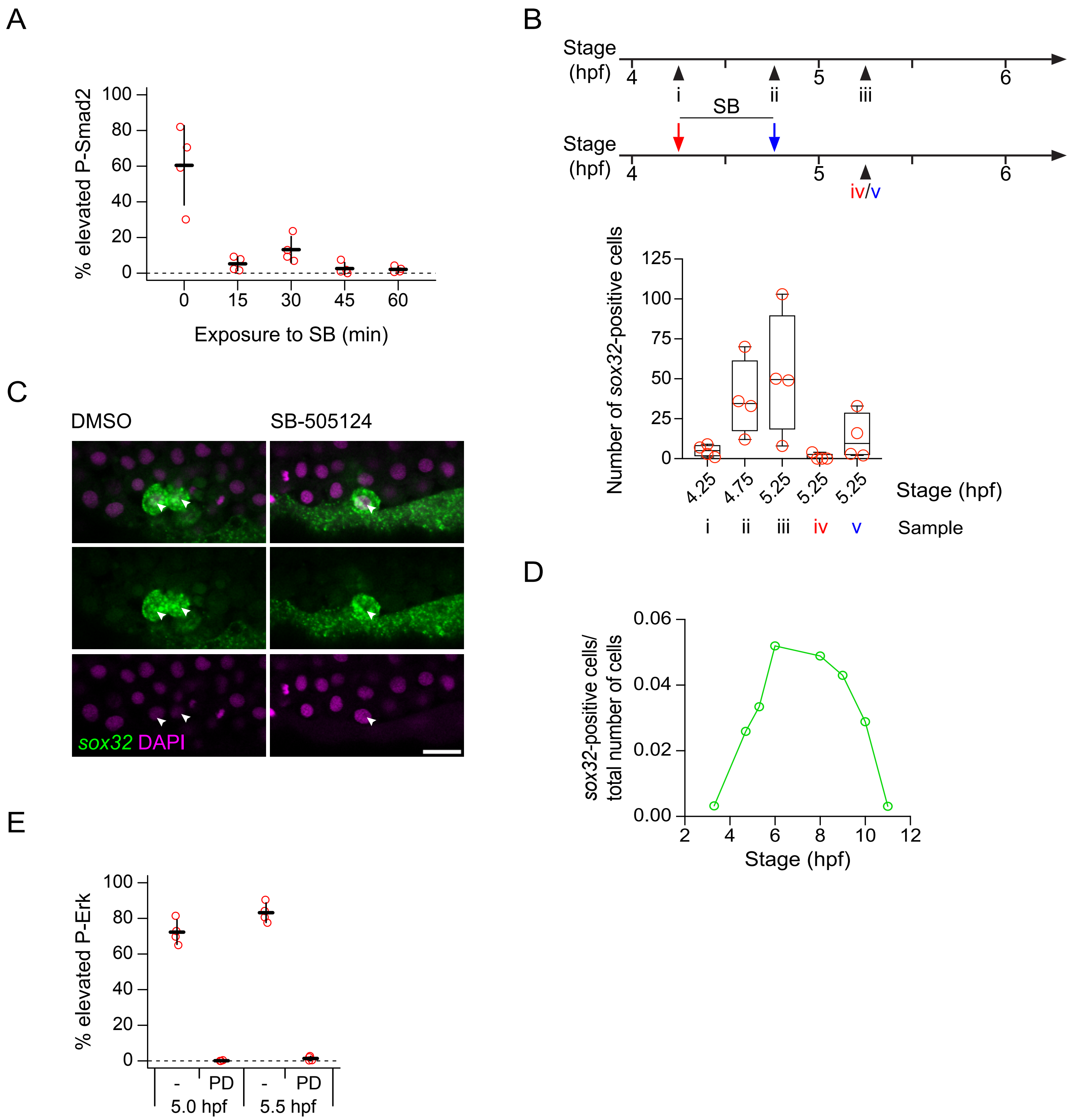

A
% elevated P-Smad2
100
80
60
40
20
0
0
15
30
45
60
Exposure to SB (min)
B
Stage
(hpf)
4
5
6
i
ii
iii
SB
iv/v
Number of sox32-positive cells
125
100
75
50
25
0
4.25
4.75
5.25
5.25
5.25
Stage (hpf)
i
ii
iii
iv
v
Sample
C
DMSO
SB-505124
sox32 DAPI
D
sox32-positive cells/
total number of cells
0.06
0.04
0.02
0.00
2
4
6
8
10
12
Stage (hpf)
E
% elevated P-Erk
100
80
60
40
20
0
-
PD
-
PD
5.0 hpf
5.5 hpf

Figure S4

Figure S4. Controls for Nodal and Fgf pathway inhibitor experiments.

(A) Plot showing percent of cells in the first two cell tiers with elevated staining intensity for P-Smad2, for 5.25 hpf embryos exposed to 10 µM SB-505124 for different periods of time. Upon 15 min exposure to the inhibitor, P-Smad2 is lost in the first two cell tiers. Elevated P-Smad2 is defined by the mean background level for all embryos, with background for each embryo defined by the 99th percentile in P-Smad2 staining intensity for the ninth and tenth cell tiers. Four embryos per dose.

(B) (Top) A scheme of the experiment is shown. (Bottom) Box and whiskers plot displaying the quantitation of *sox32*-positive cell number from the density plots shown in Figure 4C. Whiskers at min to max, box indicates SD.

(C) Single optical slices through representative *sox32*-positive cells from embryos exposed to DMSO or 10 µM SB-505124 for 45 min, showing *sox32* transcripts present in the nuclei of inhibitor-treated embryos, comparable with control embryos (arrowheads). Scalebar, 25 µm.

(D) Plot showing fraction of cells expressing > 0 read of *sox32* across gastrulation. The x axis represents developmental timing (hpf)

(E) Plot showing percent of cells in the first two cell tiers with elevated staining intensity for P-Erk, for 5.0 and 5.5 hpf embryos exposed to 5 µM PD-0325901 from 4.0 hpf. In both conditions P-Erk is lost in the first two cell tiers. Elevated P-Erk is defined by the mean background level for all embryos, with background for each embryo defined by the 99th percentile in P-Erk staining intensity for the ninth and tenth cell tiers. Four embryos per dose.

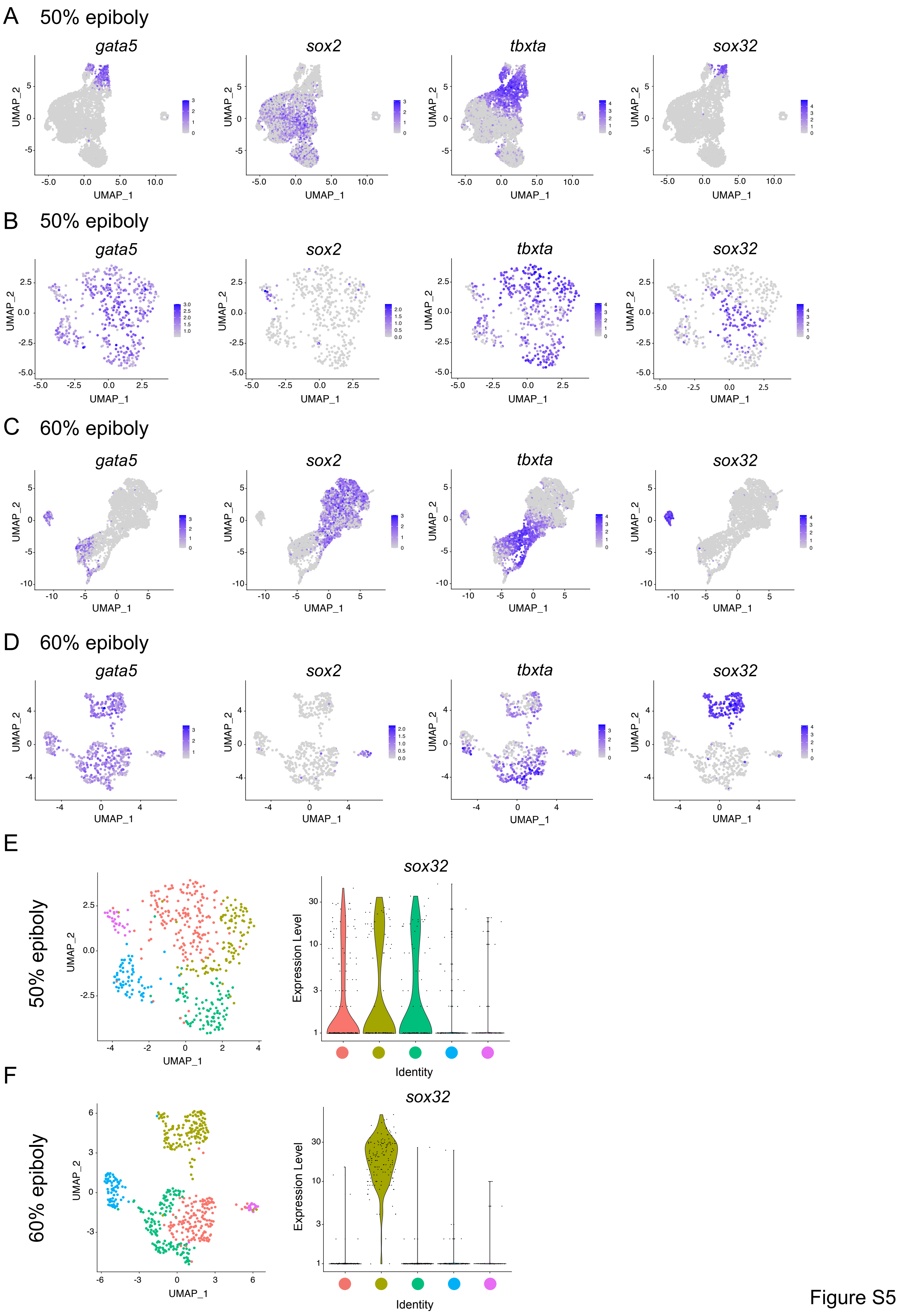
A 50% epiboly
gata5
sox2
tbxta
sox32
UMAP_1
UMAP_2
B 50% epiboly
gata5
sox2
tbxta
sox32
C 60% epiboly
gata5
sox2
tbxta
sox32
D 60% epiboly
gata5
sox2
tbxta
sox32
E
50% epiboly
sox32
Expression Level
Identity
F
60% epiboly
sox32
Expression Level
Identity

Figure S5

Figure S5. Identification of the different germ layers in scRNA-seq datasets

(A) UMAP visualization of 50% epiboly embryos showing normalized expression for (from left to right): *gata5*, *sox2*, *tbxta*, and *sox32*.

(B) UMAP visualization of *gata5*-positive cells which were isolated from the 50% epiboly sample in (A). Normalized expression for the same genes as in (A) is shown.

(C) UMAP visualization of 60% epiboly embryos showing normalized expression for (from left to right): *gata5*, *sox2*, *tbxta*, and *sox32*.

(D) UMAP visualization of *gata5*-positive cells which were isolated from the 60% epiboly sample in (C). Normalized expression for the same genes as in (C) is shown.

(E**)** UMAP visualization of *gata5*-positive cells at 50% epiboly showing five different clusters (left). Violin plot showing expression levels for *sox32* within each cluster (right). Color coding correlates expression levels with a specific cluster.

(F**)** UMAP visualization of *gata5*-positive cells at 60% epiboly showing five different clusters (left). Violin plot showing expression levels for *sox32* within each cluster (right). Color coding correlates expression levels with a specific cluster.

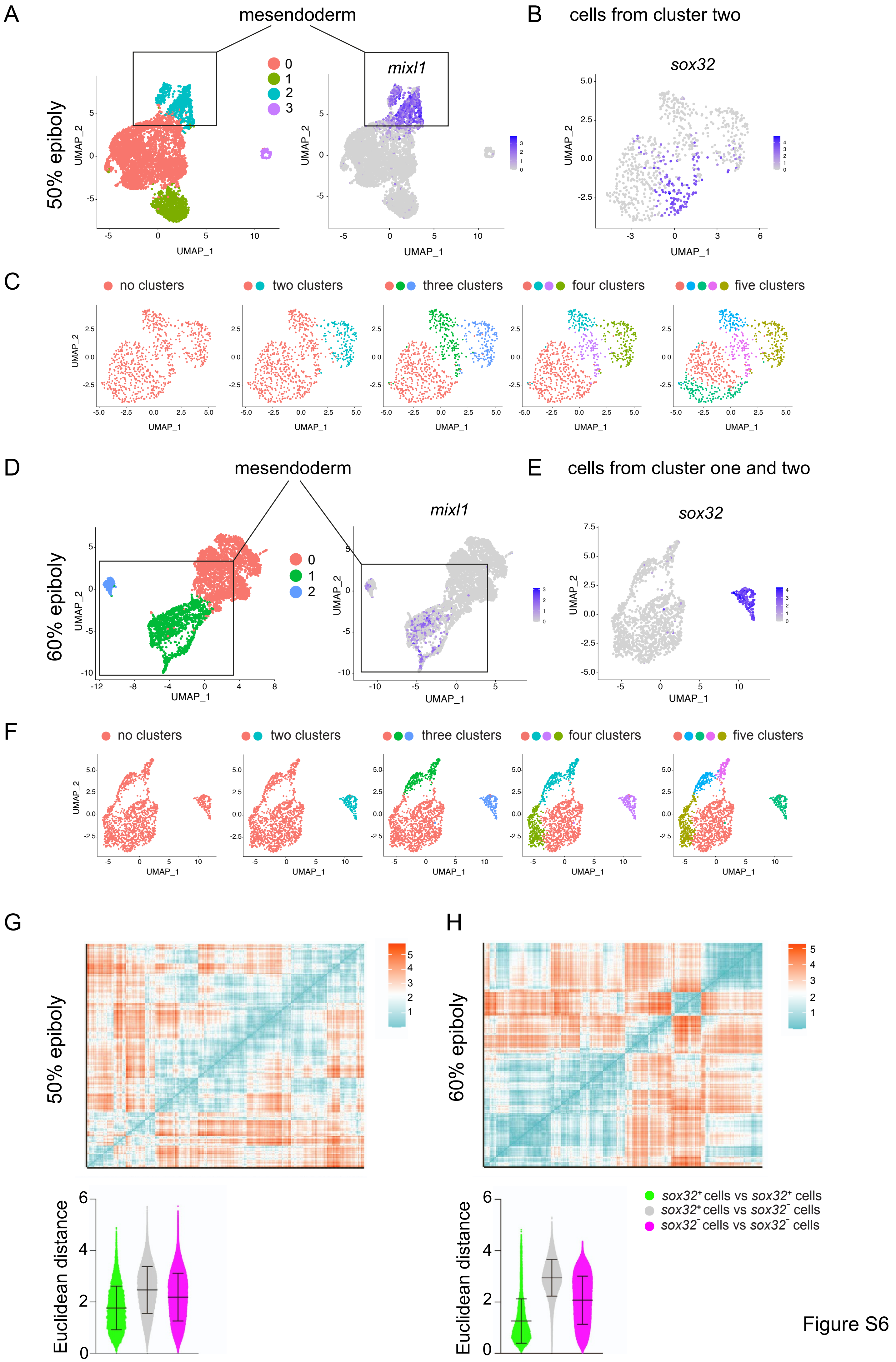

A
mesendoderm
50% epiboly
mixl1
B
cells from cluster two
sox32
C
no clusters
two clusters
three clusters
four clusters
five clusters
D
mesendoderm
60% epiboly
mixl1
E
cells from cluster one and two
sox32
F
no clusters
two clusters
three clusters
four clusters
five clusters
G
50% epiboly
Euclidean distance
H
60% epiboly
Euclidean distance
sox32+ cells vs sox32+ cells
sox32+ cells vs sox32- cells
sox32- cells vs sox32- cells
UMAP_1
UMAP_2

Figure S6

Figure S6. *sox32*-positive cells define a transcriptionally unique cluster at 60% epiboly, but not at 50% epiboly.

(A) UMAP visualization of 50% epiboly embryos showing four clusters (left). UMAP visualization showing normalized expression of *mixl1* (right). Note that cluster two corresponds to the expression of *mixl1.*

(B) UMAP visualization showing normalized expression of *sox32* within cells extracted from cluster two shown in (A)

(C) UMAP visualization of cluster two cells. Cells were clustered with increasing granularity across five iterations, Find cluster resolution = 0.05 to 0.56. Color coding refers to the different clusters. Note that *sox32*-positive cells (in B) cannot by defined by a transcriptionally distinct cluster.

(D) UMAP visualization of 60% epiboly embryos showing three clusters (left). UMAP visualization showing normalized expression of *mixl1* (right). Note that cluster one and two correspond to the expression of *mixl1.*

(E) UMAP visualization showing normalized expression of *sox32* within cells extracted from cluster one and two shown in (D)

(F) UMAP visualization of cluster one and two cells. Cells were clustered with increasing granularity across five iterations, Find cluster resolution = 0.01 to 0.2. Color coding refers to the different clusters. Note that *sox32*-positive cells in (E) define a transcriptionally distinct cluster at 60% epiboly.

(G) Top panel: distance matrix showing transcriptional distances (expressed as Euclidean distances) across 50% epiboly cells belonging to cluster two, as in (B). Bottom panel: scatter dot plot displaying cell to cell distances sorted from the comparison between *sox32*-positive vs *sox32*-positive (green) cells, *sox32*-positive vs *sox32*-negative (grey) and *sox32*-negative vs *sox32*-negative (magenta) at 50% epiboly.

(H) Top panel: as in (G) but the distance matrix shows transcriptional distances (expressed as Euclidean distances) across 60% epiboly cells belonging to cluster one and two, as in (E). Bottom panel: as in G but shows comparisons for 60% epiboly cells. Note that while at 50% epiboly cell-to-cell distance values are largely overlapping irrespective of the expression of *sox32*, at 60% epiboly *sox32*-positive cells become more transcriptionally similar to each other as they diverge, markedly, from *sox32*-negative cells.

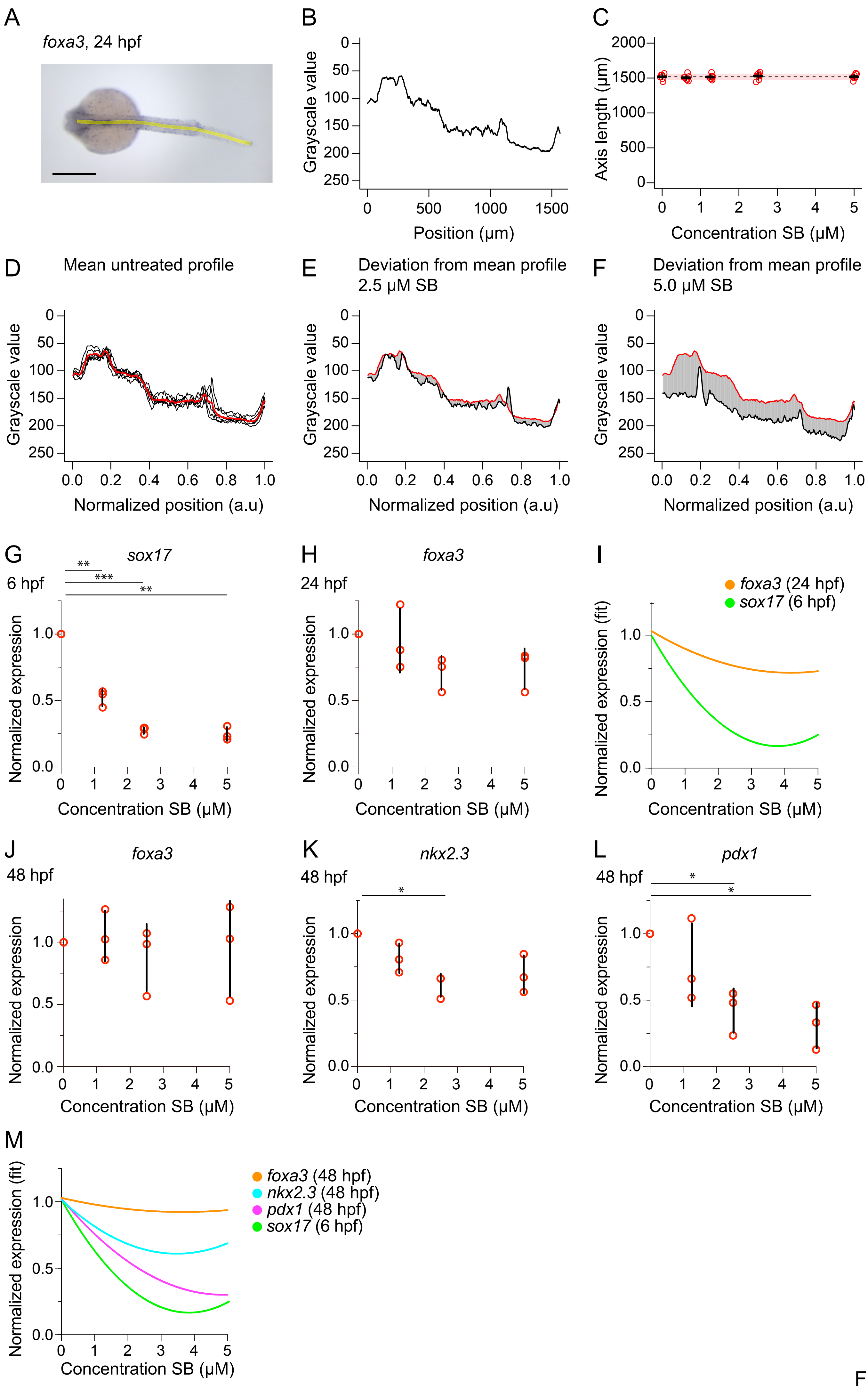
A
foxa3, 24 hpf
B
Grayscale value
Position (µm)
C
Axis length (µm)
Concentration SB (µM)
D
Mean untreated profile
Grayscale value
Normalized position (a.u)
E
Deviation from mean profile
2.5 µM SB
Normalized position (a.u)
F
Deviation from mean profile
5.0 µM SB
Normalized position (a.u)
G
sox17
6 hpf
**
***
**
Normalized expression
Concentration SB (µM)
H
foxa3
24 hpf
Normalized expression
Concentration SB (µM)
I
foxa3 (24 hpf)
sox17 (6 hpf)
Normalized expression (fit)
Concentration SB (µM)
J
foxa3
48 hpf
Normalized expression
Concentration SB (µM)
K
nkx2.3
48 hpf
*
Normalized expression
Concentration SB (µM)
L
pdx1
48 hpf
*
*
Normalized expression
Concentration SB (µM)
M
foxa3 (48 hpf)
nkx2.3 (48 hpf)
pdx1 (48 hpf)
sox17 (6 hpf)
Normalized expression (fit)
Concentration SB (µM)

Figure S7

Figure S7. Variation in initial endoderm progenitor number is rescued after gastrulation

(A) Dorsal view of 24-hpf embryo stained for *foxa3* expression by in situ hybridization. The yellow line marks an 18 µm wide strip along the axis of the embryo, along which the grayscale values of the staining are measured. Anterior to left; in lateral view dorsal is up. Scale bar, 250 µm.

(B) Grayscale intensity profile measured from embryo in (A). Anterior end of profile at 0 µm.

(C) Plot showing length of the axial profile (yellow line in (A)), for 24-hpf embryos treated at 4 hpf with different doses of SB-505124. Inhibitor treatment does not affect the gross morphology (in terms of the length of then axial profile) meaning profiles can be aligned to compare *foxa3* expression. Means ± SD are shown. Dashed line denotes untreated mean, with pink shaded area one SD.

(D) Mean intensity profile (red line) made by averaging the grayscale intensity profiles for untreated six embryos (black lines). As all embryos are approximately the same length (see (C)), profiles are normalized to the length of each embryo, averaging the intensity between profiles at 250 equally spaced points along each profile.

(E, F) Plots showing the intensity profiles for individual embryos treated with different doses of SB-505124 (black lines), relative to the mean profile (red line), with the offset between the lines shaded in grey. To calculate the difference from untreated *foxa3* intensity profiles for each embryo (plotted in Figure 7E), the grayscale value for each embryo profile is subtracted from the mean profile at 250 equally spaced points. Averaging the 250 values gives a score for each individual inhibitor-treated embryo.

(G) Plot shows qPCR for *sox17* at 6 hpf. Normalized values are shown as means ± SD. Embryos were treated with SB-505124 from sphere stage with the dose indicated on the x-axis. Zero indicates DMSO treatment. Data are the result of three biological replicates. Paired t-test. 0 vs 1.25 µM $p < 0.01$, 0 vs 2.5 µM $p < 0.001$, 0 vs 5 µM $p < 0.01$.

(H) Plot shows qPCR for *foxa3* at 24 hpf. Normalized values are shown as means ± SD. Embryos were treated with SB-505124 from sphere stage with the dose indicated on the x-axis. Zero indicates DMSO treatment. Data are the result of three biological replicates. Paired t-test.

(I) Plot shows second order polynomial fit for the expression profiles in (G) and (H).

(J) Plot shows qPCR for *foxa3* at 48 hpf. Normalized values are shown as means ± SD. Embryos were treated with SB-505124 from sphere stage with the dose indicated on the x-axis. Zero indicates DMSO treatment. Data are the result of three biological replicates. Paired t-test.

(K) Plot shows qPCR for *nkx2.3* at 48 hpf. Normalized values are shown as means ± SD. Zero indicate DMSO treatment. Embryos were treated with SB-505124 from sphere stage with the

dose indicated on the x-axis. Zero indicates DMSO treatment. Data are the result of three biological replicates. Paired t-test. 0 vs 2.5 µM $p < 0.05$.

(L) Plot shows qPCR for *pdx1* at 48 hpf. Normalized values are shown as means ± SD. Embryos were treated with SB-505124 from sphere stage with the dose indicated on the x-axis. Zero indicates DMSO treatment. Data are the result of three biological replicates. Paired t-test. 0 vs 2.5 µM $p < 0.05$, 0 vs 5 µM $p < 0.05$.

(M) Plot shows second order polynomial fit for the expression profiles in (G, J, K, L). Data are the result of three biological replicates. Paired t-test.
